# A randomised double-blind, placebo-controlled trial of pramipexole in addition to mood stabilisers for patients with treatment-resistant bipolar depression (the PAX-BD study)

**DOI:** 10.1177/02698811241309622

**Published:** 2025-01-20

**Authors:** R Hamish McAllister-Williams, Nicola Goudie, Lumbini Azim, Victoria Bartle, Michael Berger, Chrissie Butcher, Thomas Chadwick, Emily Clare, Paul Courtney, Lyndsey Dixon, Nichola Duffelen, Tony Fouweather, William Gann, John Geddes, Sumeet Gupta, Beth Hall, Timea Helter, Paul Hindmarch, Eva-Maria Holstein, Ward Lawrence, Phil Mawson, Iain McKinnon, Adam Milne, Aisling Molloy, Abigail Moore, Richard Morriss, Anisha Nakulan, Judit Simon, Daniel Smith, Bryony Stokes-Crossley, Paul RA Stokes, Andrew Swain, Adeola Taiwo, Zoë Walmsley, Christopher Weetman, Allan H Young, Stuart Watson

**Affiliations:** 1 Translational and Clinical Research Institute, Newcastle University, Newcastle upon Tyne, UK; 2 Northern Centre for Mood Disorders, Newcastle University, Newcastle upon Tyne, UK; 3 Cumbria, Northumberland, Tyne and Wear NHS Foundation Trust, Newcastle upon Tyne, UK; 4 Population Health Sciences Institute, Newcastle University, Newcastle upon Tyne, UK; 5 Department of Health Economics, Center for Public Health, Medical University of Vienna, Vienna, Austria; 6 Sheffield Health and Social Care NHS Foundation Trust, Sheffield, UK; 7 Department of Psychiatry, University of Oxford, Oxford, UK; 8 Tees, Esk and Wear Valleys NHS Foundation Trust, Darlington, UK; 9 Surrey and Borders Partnership NHS Trust, Leatherhead, UK; 10 Academic Unit of Mental Health and Neuroscience, University of Nottingham, Nottingham, UK; 11 Division of Psychiatry, Centre for Clinical Brain Sciences, University of Edinburgh, Edinburgh, UK; 12 Department of Psychological Medicine, Centre for Affective Disorders, Institute of Psychiatry, Psychology and Neuroscience, King’s College London, London, UK

**Keywords:** Pramipexole, bipolar depression, treatment resistance, randomised controlled trial

## Abstract

**Background:**

Options for ‘treatment-resistant bipolar depression’ (TRBD) are limited. Two small, short-term, trials of pramipexole suggest it might be an option.

**Aims:**

To evaluate the clinical effectiveness and safety of pramipexole in the management of TRBD.

**Methods:**

A multi-centre randomised, double-blind controlled trial including participants ⩾18 years old with TRBD (failure to respond, tolerate or clinical contraindication/patient refusal of ⩾2 of quetiapine, olanzapine, lamotrigine or lurasidone) randomised 1:1 to pramipexole (max 2.5 mg/day salt weight) or placebo added to ongoing mood stabiliser (*n* = 39). Primary outcome: Quick Inventory of Depressive Symptoms, Self-rated (QIDS-SR) at 12 weeks. Up to 48 weeks follow-up.

**Results:**

Pramipexole (*n* = 18) was associated with a greater reduction in QIDS-SR score at 12 weeks versus placebo (*n* = 21, 4.4 (4.8) vs 2.1 (5.1)): a medium sized (*d* = −0.72) but not statistically significant difference (95% CI: −0.4 to 6.3, *p* = 0.087). Similarly, there was a non-significant approximate 2-point (*d* = −0.76) improvement in pleasure at 6 weeks (95% CI: −0.11 to 4.20). Significant advantages of pramipexole on QIDS-SR score (6.28 points: 95% CI: 1.85–10.71) and psychosocial function (5.36 points: 95% CI: 0.38–10.35) were seen at 36 weeks post-randomisation, and on the response (46% vs 6%; *p* = 0.026) and remission (31% vs 0%; *p* = 0.030) rates at trial exit (48 weeks or last available data after 16 weeks for those affected by the early study closure). Hypomania ratings were significantly higher at 12 weeks. Otherwise, pramipexole was well tolerated.

**Conclusions:**

Clinically large, but statistically non-significant, effects of pramipexole on depression at 12 weeks, with significant longer-term benefits on mood and function were observed. Pramipexole use was complicated by dose titration and increased hypomanic symptoms. The small sample size limits interpretation. Furthermore, larger randomised placebo-controlled trials are warranted.

## Introduction

Patients with bipolar disorder (BD) are symptomatic around 50% of the time, the vast majority of which is depression (Judd et al., 2002, 2003). In the UK, the National Institute for Health and Care Excellence (NICE) guidelines list three treatments for bipolar depression: lamotrigine, quetiapine, and olanzapine (with or without fluoxetine; NICE, 2014), with the latter two poorly tolerated due to weight gain and sedation (Calabrese et al., 2005; Tohen et al., 2003). Around 35% of patients with BD who are currently depressed in the UK take at least one antidepressant (POMH-UK, 2018) despite evidence that they lack efficacy (NICE, 2014). This demonstrates the clinical challenge posed by treatment-resistant bipolar depression and that many people with BD experience treatment-resistant bipolar depression (TRBD). The prevalence of TRBD is unknown due to a lack of a widely used consensus definition. However, around 50% of people with bipolar depression remain depressed at 6 months, and 30% at a year because of non-response, intolerance, or non-acceptance, of treatment (Kupfer et al., 2000). As a result, TRBD is the major contributor to the enormous burden of disease associated with BD, which in the UK is estimated to have an annual cost of around £15,000 per patient and a population-level economic burden of £6.43 billion from 2018 to 2019 prices of which 30.5% is direct healthcare costs (Simon et al., 2021).

Pramipexol is a dopamine D_2_, D_3_, and D_4_ agonist with its highest affinity and greatest potency for D3 receptors (Deutschländer et al., 2016; Kvernmo et al., 2006; Mierau et al., 1995). Its potential role as a treatment for depressive episodes in BD is supported by pre-clinical investigations (Chiu et al., 2015; Kitagawa et al., 2009; Willner et al., 1994), including increased hippocampal neurogenesis (Chiu et al., 2015), an effect believed to be common to antidepressants (Duman et al., 2001; Serafini et al., 2014). Pramipexole has extensive evidence for efficacy in Parkinson’s disease (Shen et al., 2017), for which it has a marketing licence in Europe and the United States. A meta-analysis of pramipexole in Parkinson’s disease reported improvement in depressive symptoms (Leentjens et al., 2009). A subsequent 12-week randomised double-blind placebo-controlled trial of pramipexole in participants with Parkinson’s disease and significant depressive symptoms reported a significant benefit on the mood that was independent of any motor improvements seen (Barone et al., 2010). Such findings, together with hypothesised roles for a hypo-dopaminergic state underlying bipolar depression (Ashok et al., 2017) and naturalistic and open-label data (Dell’osso et al., 2013; El-Mallakh et al., 2010; Fawcett et al., 2016; Hori and Kunugi, 2012; Lattanzi et al., 2002; Perugi et al., 2001; Sporn et al., 2000) led to two small pilot randomised placebo-controlled trials (RCTs) which both reported positive findings (Goldberg et al., 2004; Zarate et al., 2004).

The PAX-BD trial aimed to evaluate the clinical effectiveness and safety of pramipexole versus placebo alongside standard mood stabilising medication in the management of patients with TRBD. The trial was intended to be an appropriately powered, pragmatic, RCT with economic evaluation (published separately), with the primary outcome at 12 weeks post-randomisation and a total of 48 weeks of follow-up. The hypothesis was that pramipexole would lead to a significantly greater reduction in depressive symptoms at 12 weeks from baseline compared with placebo. Additional study aims were to examine the effect of pramipexole over 48 weeks on mood, anxiety and psychosocial function, and assess the safety and tolerability of treatment.

The PAX-BD study was terminated early by the funder due to slow recruitment, in part related to the COVID-19 pandemic. As a result, the final study population was well short of that defined by the original power calculation. The findings of the study are therefore presented here, together with a post hoc power calculation to determine the sample size required for a future definitive trial.

## Methods

The study was a phase III multi-centre, randomised, double-blind, placebo-controlled, parallel-group, trial, funded by the UK National Institute for Health Research (NIHR) Health Technology Appraisal (HTA) Panel. It included two stages: pre-randomisation and randomisation. The purpose of the pre-randomisation stage was to prepare participants for recruitment to the randomisation stage by adjusting antipsychotics (where required), commencing mood stabilising medication (where required) and ensuring participant engagement with the TrueColours platform for collecting self-report rating scale data and the central Research Assistant (RAs) team. TrueColours is an online self-management system, hosted by Oxford Health NHS Foundation Trusts, that allows individuals to rate their symptoms using a range of self-completed rating scales. For PAX-BD, participants were requested to complete the Quick Inventory of Depressive Symptoms – Self-rated (QIDS-SR; Rush et al., 2003), Altman Self-rating Scale of Mania (ASRM) (Altman et al., 1997) and Generalised Anxiety Disorder 7 (GAD-7; Spitzer et al., 2006) scales on a weekly basis. In the randomisation stage, participants were randomised on a 1:1 basis to receive either pramipexole or matched placebo alongside their existing mood stabilising medication, in a double-blind fashion.

The methods and study protocol for the PAX-BD study have been previously published (Azim et al., 2021).

### Sample size

The original sample size calculation described in the protocol paper (Azim et al., 2021) was based on a 3-point difference in QIDS-SR (equates to Cohen’s *d* = 0.4 which is considered clinically meaningful) between the drug and placebo (at *p* < 0.05) and drop-out rates of 30% during the pre-randomisation stage (BALANCE investigators and collaborators et al., 2010) and 20% between randomisation and 12 weeks (Geddes et al., 2016) that were seen in previous UK studies in similar populations. This estimated the need for a sample size of 290 at randomisation and 414 at the point of recruitment to pre-randomisation.

### Participant identification

Participants were opportunistically identified from within the patient populations under the care of secondary mental healthcare services in one of 14 participating secondary care mental health NHS Trusts across the UK. Study activity was conducted at these sites with assessments conducted remotely online and by phone with RAs based at the study sponsor site: Cumbria, Northumberland, Tyne and Wear (CNTW) NHS Foundation Trust (Supplemental Table S1).

Written informed consent was received by General Medical Council registered medical practitioners for all participants. Participants met the criteria for having TRBD. In this context, a ‘failed’ course of treatment in this study, was defined as a clinically determined inadequate response to an ‘adequate trial’ at an ‘adequate dose’ (both operationally defined based on current guidance), or an inability to tolerate or accept treatment. This reflects the current point of clinical equipoise, supported by our patient and public involvement (PPI) group. Due to the limited number of current treatment options available for TRBD, it defines a point at which there is potential for significant change to clinical and cost trajectories.

The full inclusion criteria for entering the pre-randomisation stage were as follows:

- Aged over 18 years.
- Under the care of secondary care mental health services.
- Had a DSM-5 diagnosis of BD (type I or II).
- Currently depressed with a QIDS-SR > 10.
- A decision had been made that a change in medication was clinically indicated.
- The current episode of depression had failed to respond to adequate treatment trials, or there was a lack of tolerability, or the patient refused, or there was a contraindication of two different medications from a list of quetiapine, olanzapine (±fluoxetine) lamotrigine or lurasidone. These four treatments were chosen for this criteria based on the first three being the only specific options recommended for the treatment of bipolar depression by the UK NICE (NICE, 2014) and the fourth being an additional option included in the bipolar guidelines produced by the British Association for Psychopharmacology (BAP: Goodwin et al., 2016).
- If female and of child-bearing potential, participants had to have had a negative pregnancy test (urine beta-human chorionic gonadotropin (β-hCG)) and were required to use a highly effective contraceptive method for the duration of the trial.

Participants were excluded if they met the following criteria:

- A diagnosis of a severe substance use disorder.
- Current psychotic symptoms.
- A history of retinal disease, renal disease, current cardiovascular symptoms, Parkinson’s disease or restless leg syndrome.
- The clinical concern of previous impulse control behaviours.

Participants were eligible to proceed to the randomisation stage after a minimum of 23 days (4 weeks excluding the final weekend and allowing for 3 days flexibility) in the pre-randomisation stage if they continued to meet pre-randomisation eligibility criteria and had engaged with study procedures. In addition, participants needed to be taking at least one mood stabilising medication (lithium, valproate, carbamazepine, lamotrigine – commenced during pre-randomisation if necessary), and all regular psychotropic medication, including mood stabilisers, had to be at a stable dose for a minimum of 4 weeks. They were excluded if they had experienced psychotic symptoms over the preceding 4 weeks; there had been any deterioration in physical or mental health since pre-randomisation; or they had started, or planned to start, specific psychotherapy from 4 weeks before randomisation through to week 12 post-randomisation.

At the study commencement, participants were also ineligible for randomisation if they were taking an antipsychotic, which was withdrawn during pre-randomisation if necessary. This was a requirement of the funder on the basis that pramipexol is a dopamine agonist and antipsychotics are dopamine antagonists. It severely limited recruitment. Midway through this requirement was adjusted to reflect a more ‘real-world’ situation, supported by the study’s PPI group. The study funder and ethics committee approved an amendment to allow participants to continue on an antipsychotic post-randomisation as long as it was being used at a dose less than a stated maximum. These maximum doses were defined based on the relative D2/D3 receptor affinity of the drug. Details can be found, along with full pre-randomisation and post-randomisation criteria, in the published study protocol (Azim et al., 2021) and Supplemental Tables S2 and S3.

### Randomisation and trial medication

Following additional written informed consent, eligible participants were randomised 1:1 using a central, secure, 24-h web-based system, Sealed Envelope™, to receive either pramipexole or a visually identically matched placebo, with participants, clinicians and the research study team blinded to treatment allocation. Placebo was manufactured and delivered blinded along with pramipexole by MODEPHARMA, UK. A non-deterministic minimisation algorithm was used to produce treatment groups balanced for important prognostic factors. The first 10 patients were allocated randomly without minimisation to avoid predictability. The algorithm minimised for nine variables related to prognosis at baseline: bipolar (I vs II); severity of depression; age (18–50, >50); sex; recruitment site region; baseline mood stabiliser; whether the participant was taking an antidepressant; whether an antipsychotic had been withdrawn at baseline (subsequently changed to whether on one or not at randomisation); and the number of mood episodes in the past year (<4 and ⩾4). At each randomisation, the system calculated the imbalance between the minimisation factors and allocated the treatment group that best restored the balance. The system incorporated a 20% ‘random element’ in that for 20% of cases the system ignored the balance between the minimisation factors and allocated treatment groups based on chance to avoid predictability based upon past allocations.

Participants received trial medication supplied by post from a central pharmacy at the CNTW Trust for up to 52 weeks. Participants self-administered trial medication. Adherence was checked verbally by the study RAs during regular phone contacts and recorded by participants in study diaries. There was an initial 4-week titration phase with increasing doses of 0.25 mg/day every 3 days to a target of 2.5 mg/day dependent on tolerability and response (based on the mean dose achieved in a case series of patients with unipolar or BD who had responded or remitted with pramipexole: Fawcett et al., 2016). The dose achieved was then fixed through weeks 5–12. After the primary outcome time point, the dose could be adjusted flexibly to between 0.25 and 2.5 mg/day (determined by response and tolerability) with all other medication kept stable (unless clinical need dictated otherwise). Mood stabilisers could also be adjusted during this phase but while the participant remained on trial medication it was recommended that they also remain on at least one of the four recommended mood stabilisers. At the end of participation (either end of trial or early withdrawal), medication was tapered at a rate of 0.25 mg every 3 days. All medication weights reported are salt weights.

### Outcomes

The schedule of events is reported in the study protocol paper (Azim et al., 2021) and detailed in the Statistical Analysis Plan (SAP – see Supplemental material). The primary outcome was the change in QIDS-SR score at 12 weeks compared with baseline at the time of starting the study medication. Secondary efficacy outcomes included the following:

- QIDS-SR and GAD-7 scores were rated weekly from baseline to 48 weeks.
- Change in QIDS-SR score from baseline at 24, 36 and 48 weeks.
- Response (defined as ⩾50% reduction in QIDS-SR score from baseline) and remission (QIDS-SR score ⩽5) rates at 12, 24, 36 and 48 weeks.
- Snaith–Hamilton Pleasure Scale (SHAPS; Snaith et al., 1995) at baseline and weeks 6 and 12.
- Work and Social Adjustment Scale (WSAS; Mundt et al., 2002) at weeks 6, 12, 24, 36 and 48, compared to baseline.

Secondary safety and acceptability outcomes included the following:

- Assessment of manic symptoms using the ASRM completed weekly.
- Rates of impulsivity using the Questionnaire for Impulsive-Compulsive Disorders in Parkinson’s Disease – Rating Scale (QUIP-RS; Weintraub et al., 2012) at baseline and weeks 6, 12 and 4 weekly thereafter.
- Side effects and overall acceptability using the Treatment Satisfaction Questionnaire for Medication (TSQM; Atkinson et al., 2004) at weeks 6, 12 and then 4 weekly thereafter.
- Rates of adverse events (AEs), serious adverse events (SAEs) and suspected unexpected serious adverse reactions (SUSARs).

For comparison with other studies, the observer rated Montgomery Asberg Depression Rating scale (MADRS) (Montgomery and Asberg, 1979), Quick Inventory of Depressive Symptoms – clinician rated (QIDS-C; Rush et al., 2003) and Young Mania Self-Rating Scale (YMRS; Young et al., 1978) were administered at baseline and week 12 via telephone by the central RA team.

There were no changes to outcome assessments after the study’s commencement.

### Patient and public involvement

The trial involved a group of people with lived experience of bipolar and ‘regular’ depression (PPI group) from the initial question design. Members of the PPI group met numerous times during the trial and were involved in reviewing patient documents and discussing ethical issues around recruiting and retaining participants in the trial. The group assisted in training Research Assistants prior to them being cleared to have contact with participants. Changes based on PPI input were made to the trial design to improve the patient experience. The PPI group chair attended and provided input into Trial Management Group and core-team catch-up meetings, with PPIE a standing item on the agenda. The group reviewed the reporting for the trial which led to writing a paper evaluating the impact of the PPI within this trial (published elsewhere).

### Statistical analysis

The analysis was governed by an a priori Statistical Analysis Plan (SAP: see Supplemental material). The primary outcome measure was the QIDS-SR score 12 weeks post-randomisation analysed using an analysis of covariance (ANCOVA) comparing scores between the treatment groups while adjusting for QIDS-SR score at the time of randomisation (week 0), using the intention to treat (ITT) population where all participants, including any later determined to have been ineligible or protocol violators, were included in the analysis with participants retained in their randomised treatment group. As defined in the SAP, there had been an intention to undertake additional analysis of the primary outcome measure covarying for randomisation minimisation factors and any baseline characteristics found in exploratory univariate analyses to be related to the outcome. However, given the small sample size due to the early trial closure, these analyses were not undertaken.

Secondary analyses focused on the completer populations defined as those participants providing data at specified time points. ANCOVA analysis, covarying for baseline values was conducted for QIDS-SR, GAD-7, WSAS, ASRM and QUIP-RS scores at the protocol and SAP-specified 12- and 48-week follow-up time points. Following observations of the weekly QIDS-SR scores, additional analysis of QIDS-SR GAD-7 and ASRM was conducted at ad hoc 24 and36 week time points post-randomisation when WSAS and QUIP-RS analyses were already pre-specified in the SAP. In addition, as specified in the protocol and SAP, SHAPS was analysed at 6 and 12 weeks and MADRS, QIDS-C and YMRS scores at 12 weeks only. Response and remission rates were compared between treatment arms using Fisher’s Exact test.

A two-sided significance level of *p* < 0.05 was used, though other than for the primary outcome analysis and when using a Fisher’s Exact test, 95% confidence intervals rather than *p*-values are reported.

As a result of the early closure of the trial, many of those contributing 12-week data were not able to supply data for later time points. It was agreed with the Trial Steering Committee to redefine trial exit as the 48-week time point or the last available data after 16 weeks for those affected by the early study closure.

All analyses were conducted with STATA version 16 (StataCorp LLC, Texas, USA).

### Ethics and governance

The sponsor for the trial was Cumbria, Northumberland Tyne and Wear NHS Foundation Trust (reference RES-17-031). Clinical Trial Authorisation was received from the UK Medicines and Health Care Regulatory Authority (MHRA) (CTA reference: CTA 31857/0003/001-0001). A Favourable Opinion was granted by the North East, Newcastle and North Tyneside 2 Research Ethics Committee (REC reference 19/NE/0233).

PAX-BD was registered as ISRCTN72151939 and EudraCT 2018-2869-18.

## Results

### Recruitment and trial population

**Figure 1.**
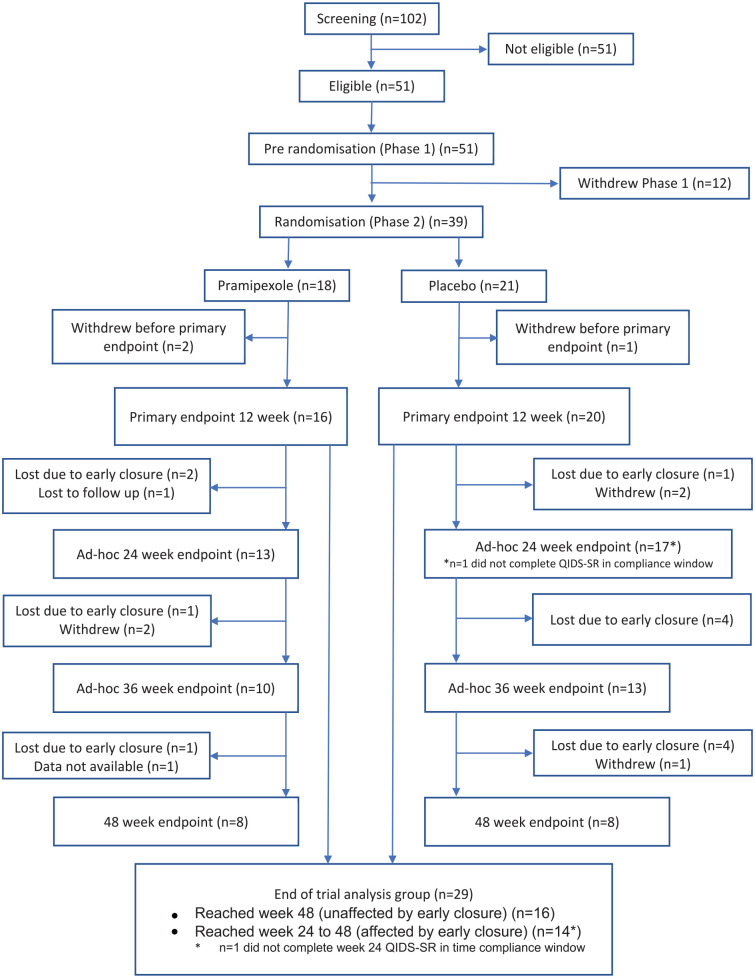
CONSORT diagram illustrating the flow of participants through the trial. As indicated by the ‘*’ symbol (see the numbers for the ad-hoc 24 week time point and end of trial analysis group), one participant in the placebo arm did not complete the 24 week QIDS-SR in the time compliance window.

Fourteen UK NHS sites recruited participants into the pre-randomisation phase, with participants from 12 being randomised. Recruitment and participant flow through the trial are reported in the CONSORT diagram (Figure 1). The recruitment period ran between 28th November 2019 and 5th April 2022 to the pre-randomisation stage, but recruitment was suspended between 18th March and 25th October 2020 due to the COVID pandemic. Following the re-opening of the study, recruitment was slower than anticipated due to research infrastructure being diverted to COVID-related research and the impact the pandemic had on the way mental health services were provided with more remote contact and less opportunity to approach potential participants. The trial was prematurely closed due to slow recruitment at which point, on the advice of the PPI group to minimise the negative impact on participants, all those recruited to pre-randomisation were given the opportunity to complete the stage and, if eligible, be randomised and followed up to at least the 12-week primary outcome time point or for as long as the trial continued to run. The last participant was randomised on 14th June 2022 and the last participant, last visit took place on 29th October 2022.

A total of 102 patients were assessed for entry into the trial. Fifty percent (51 participants) were found to be eligible and entered pre-randomisation. Demographic and illness characteristics are shown in Table 1. Most participants had a diagnosis of BD type I (73%) with a mean age of onset of 28.4 years and the duration of the current episode of 27.5 months. For just over half (51%), this was the only episode of BD in the last year, while 8 (16%) participants met the criteria for rapid cycling BD with ⩾4 episodes in the last 12 months (Barrios et al., 2001). On entry to the trial, 42 (82%) were on a mood stabiliser, 29 (57%) on an antipsychotic, 29 (57%) on an antidepressant, 25 (49%) on an anxiolytic/hypnotic, 10 (20%) on another psychotropic and 33 (65%) on a non-psychotropic (specific drugs and doses in Supplemental Table S4). Ten (20%) were on two mood stabilisers and 29 (57%) were on an antidepressant. TRBD was defined in terms of medication non-response, intolerance or declined/not clinically indicated. The most common reason for participants to meet the TRBD criteria was due to lack of tolerability or declined or felt to be clinically inappropriate (see Supplemental Table S5). The majority of participants had failed to respond to no or one medication in the current episode (65%), and only a small minority had failed to respond to 3 or 4 medications (6%: see Supplemental Table S6).

**Table 1.** Demographics, illness characteristics and medication of participants recruited into the trial. Data are reported for those who entered the pre-randomisation stage but did not progress to randomisation, and those who were randomised broken down by treatment arm.

Demographic	Units/statistics	Pre-randomisation: did not progress (*n* = 12)	ITT population (*n* = 39)	
			Pramipexole (*n* = 18)	Placebo (*n* = 21)
Gender	Male	3 (25%)	10 (56%)	11 (52%)
	Female	9 (75%)	8 (44%)	10 (48%)
Age (years)	Median (LQ, UQ)	45 (41, 56)	49 (40, 57)	49 (36, 58)
	Mean (SD)	47.5 (12.2)	47.7 (9.8)	47.6 (12.6)
	Range (min, max)	25, 66	30, 61	26, 68
BMI	Median (LQ, UQ)	31.4 (24.8, 44.3)	30.1 (24.8, 39.6)	29.3 (25.6, 32.3)
	Mean (SD)	34.6 (12.3)	34.2 (13.9)	30.5 (9.1)
	Range (min, max)	20.3, 55.4	20.0, 78.0	21.2, 63.3
Current smoker	yes	2 (17%)	5 (28%)	5 (24%)
	no	6 (50%)	13 (72%)	16 (76%)
	Not recorded	4 (33%)	0	0
Max education level	GSE, GCSE, O-level	2 (17%)	4 (22%)	2 (10%)
	A levels or equivalent	0 (0%)	2 (11%)	6 (29%)
	Undergrad. degree	1 (13%)	6 (33%)	4 (19%)
	Postgraduate degree	2 (17%)	4 (22%)	3 (14%)
	None/other/prefer not to answer/not applicable/don’t know	3 (25%)	2 (11%)	6 (29%)
Bipolar type	I	9 (75%)	13 (72%)	15 (71%)
	II	3 (25%)	5 (28%)	6 (29%)
Age at 1st onset (years)	Median (LQ, UQ)	34 (18, 48)	27 (21, 33)	23 (17, 31)
	Mean (SD)	33.9 (15.0)	27.7 (9.9)	25.8 (12.4)
	Range (min, max)	13, 61	14, 48	9, 59
Duration of the current episode (months)	Median (LQ, UQ)	16 (7, 20)	8 (3, 42)	18 (7, 41)
	Mean (SD)	17.7 (14.9)	25.3 (33.1)	35.0 (47.3)
	Range (min, max)	2, 49	1, 112	2, 179
Number of mood episodes in last 12 months	<4	11 (92%)	15 (83%)	17 (81%)
	⩾4	1 (8%)	3 (17%)	4 (19%)
QIDS-SR	Median (IQR)		15.5 (12, 19)	18 (16, 21)
	Mean (SD)		15.2 (5.0)	17.6 (4.8)
	Range (min, max)		3, 24	9, 25
GAD-7	Median (IQR)		9 (6, 12)	10 (5, 18)
	Mean (SD)		9.2 (5.3)	11.4 (6.7)
	Range (min, max)		0, 21	3, 21
SHAPS^a^	Median (IQR)		6 (3, 10)	7.5 (5, 10.5)
	Mean (SD)		6.2 (4.3)	7.7 (3.6)
	Range (min, max)		0, 13	1, 14
WSAS^b^	Median (IQR)		30.5 (25.5, 34)	32 (24.5, 35.5)
	Mean (SD)		29.3 (6.8)	30.0 (7.1)
	Range (min, max)		15, 38	17, 40
ASRM	Median (IQR)		0 (0, 0)	0 (0, 0)
	Mean (SD)		0.5 (1.2)	0.2 (0.5)
	Range (min, max)		0, 5	0, 2

a Two baseline SHAPS scores missing: Pramipexole *n* = 17, placebo *n* = 20.

b Three baseline WSAS missing: Pramipexole *n* = 16, placebo *n* = 20.

Twelve participants recruited into the pre-randomisation stage (23.5%) did not progress to randomisation. Reasons for lack of continuation to randomisation were varied (death (1), ineligible for randomisation (3: two due to no longer meeting criteria for a current episode of depression and one due to a relapse in alcohol use), unable to tolerate withdrawal from an antipsychotic (3), COVID infection (2), study closedown (1), other reasons not disclosed (2)). There was little difference between the time in pre-randomisation between those randomised to pramipexole and those randomised to placebo.

Thirty-nine participants were randomised with the primary analysis for the trial carried out on this ITT population. Three withdrew prior to the primary outcome time point: two in the pramipexole arm (who withdrew at 3 and 6 weeks) and one in the placebo treatment arm (who withdrew at 6 weeks). As can be seen from Table 1, there were few differences in participant characteristics between treatment arms. Note that the baseline QIDS-SR scores, as well as other key questionnaire scores, were not balanced between treatment arms. This is likely due to the small sample size.

All participants were taking a mood stabiliser at randomisation, the most common being lithium and valproate (see Supplemental Table S4). The vast majority (90%) were on the same medication at the same dose as at the point of recruitment to pre-randomisation. At the point of randomisation, 18 (46%) participants were taking an antipsychotic and 56% taking an antidepressant.

### Study medication received

Of the 39 participants in the ITT sample, 18 received pramipexole and 21 placebo. The dose of medication achieved at the end of the titration phase and used for the fixed-dose period was 2.18 mg/day (SD: 0.58; range: 0.75–2.50) for the pramipexole arm and 2.25 mg/day (SD: 0.55; range: 0.50–2.50) for the placebo arm. By the end of the flexible-dose phase, the doses were 2.02 mg (SD: 0.64; range: 0.25–2.50) for pramipexole and 1.66 mg (SD: 0.55; range: 0.25–2.50) for placebo.

Nine participants (50%) in the pramipexole group and six (29%) in the placebo group recorded in their diaries that they had missed at least one dose of trial medication.

### Outcomes

There was a high rate of completion by participants of the online weekly rated scales across their entire participation in the trial of up to 48 weeks: 78% for the QIDS-SR, 80% for ASRM and 81% for GAD-7 across both treatment arms. Weekly average scores with 95% CI for the QIDS-SR and ASRM are shown in Supplemental Figure S1.

A summary of the outcomes from the study is shown in Table 2.

**Table 2.** Summary of PAX-BD study primary and main secondary outcomes.

Outcome measure		6 weeks	12 weeks	24 weeks	36 weeks	48 weeks	Exit from study
Numbers of participants contributing data (unless otherwise stated)	Pramipexole	17	16	13	9	8	13
	Placebo	21	20	17	10	8	16
QIDS-SR			2.9 (−0.4 to 6.3)**	1.19 (−2.28 to 4.65)	**6.3 (1.85–10.71)**	5.5 (−0.045 to 11.0)	
QIDS-SR response rate (%) pramipexole vs placebo			25% vs 15%				**46% vs 6%**
QIDS-SR remission rate (%) pramipexole vs placebo			12.5% vs 15%				**31% vs 0%**
MADRS			2.59 (−4.54 to 9.72) PAX = 16; Plac = 19				
QIDS-C			2.30 (−1.72 to 6.32) PAX = 16; Plac = 19				
GAD-7			1.32 (−0.87 to 3.50)	1.77 (−2.04 to 5.59)	3.44 (−1.06 to 6.99)	0.18 (−6.05 to 6.41)	
SHAPS		2.04 (−0.11 to 4.20) PAX = 14; Plac = 18	0.76 (−1.78 to 3.29) PAX = 15; Plac = 19				
WSAS			0.99 (−4.16 to 6.14) PAX = 15; Plac = 17	2.21 (−4.27 to 8.69) PAX = 10; Plac = 15	**5.36 (0.38–10.34)** PAX = 8; Plac = 9	**9.63 (0.25–19.02)** PAX = 7; Plac = 5	
ASRM			**1.2 (0.02–2.42)**	0.92 (−1.09 to 2.93)	0.98 (−0.37 to 2.35)	0.56 (−1.55 to 2.67)	
YMRS			1.16 (−1.09 to 3.41) PAX = 16; Plac = 19				
QUIP-RS		0.65 (−9.01 to 10.31) PAX = 13; Plac = 20	2.71 (−6.17 to 11.59) PAX = 13; Plac = 18	3.79 (−5.15 to 12.73) PAX = 12; Plac = 16	−3.04 (−18.57 to 12.48) PAX = 8; Plac = 9	5.15 (−7.22 to 17.52) PAX = 8; Plac = 6	
TSQM		19.17 (5.21–33.13) PAX = 14; Plac = 20	6.17 (−14.86 to 27.20) PAX = 12; Plac = 17	5.73 (−15.07 to 26.53) PAX = 11; Plac = 16	21.83 (−10.42 to 54.07) PAX = 7; Plac = 9	15.48 (−13.86 to 44.81) PAX = 8; Plac = 7	

Results presented (unless otherwise indicated) as the difference in scores between treatment arms together with 95% confidence intervals. All differences in favour of pramipexole (i.e. less depression, less anxiety, more pleasure, better social adjustment, better treatment satisfaction) compared with placebo except hypomanic symptoms and impulse control behaviours (greater with pramipexole), and QIDS-SR remission rate at 12 weeks. Results in bold indicate a statistically significant difference between treatment arms at *p* < 0.05.

ASRM: Altman self-rating scale of mania; GAD-7: generalised anxiety disorder 7 item scale; MADRS: Montgomery Asberg depression rating scale; PAX: pramipexole (referring to a number of participants in treatment arm); Plac: placebo (referring to a number of participants in treatment arm); QIDS-C: quick inventory of depressive symptoms, clinician-rated; QIDS-SR: quick inventory of depressive symptoms, self-rated; QUIP-RS: questionnaire for impulsive-compulsive disorders in Parkinson’s disease – rating scale; SHAPS: Snaith–Hamilton pleasure scale; TSQM: treatment satisfaction questionnaire for medication; WSAS: work and social adjustment scale; YMRS: young mania self-rating scale.

** Primary outcome.

### Primary outcome

The decrease in QIDS-SR from baseline to 12 weeks was over twice as great as in the placebo arm with means (SDs) of −4.4 (4.8) and −2.1 (5.1), respectively. Adjusted for the effects of baseline QIDS-SR, the score at 12 weeks was 2.9 points lower for participants randomised to pramipexole (95% CI: −0.4 to 6.3, *p* = 0.0865), with a Cohens *d* effect size of 0.72. None of the minimisation or baseline variables had a statistically significant influence on this.

### Secondary outcomes

**Figure 2.**
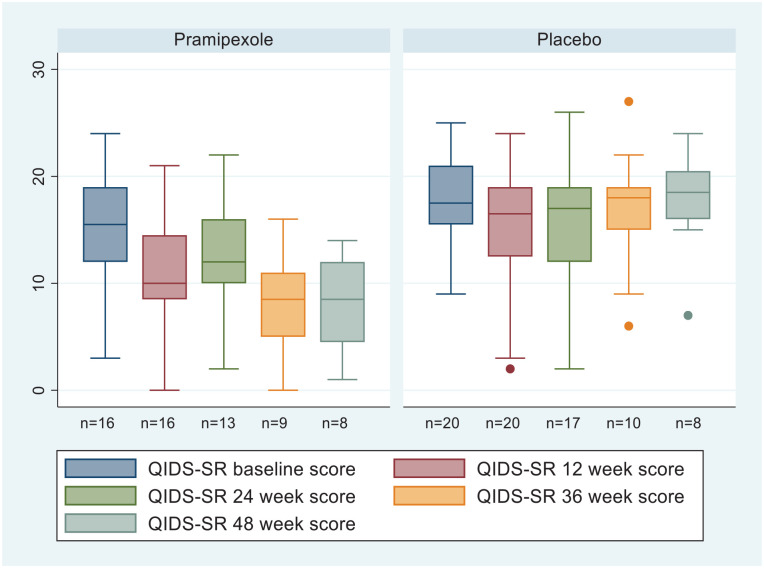
Boxplots of QIDS-SR scores for completers at baseline, 12, 24, 36 and 48 weeks.

Completer analyses of QIDS-SR scores at 12, 24, 36 and 48 weeks including those participants still in the trial at these times (see Figure 2) showed differences of 6.3 points (95% CI: 1.85–10.71) greater reduction for pramipexole compared with placebo at 36 weeks and 5.5 points (95% CI: −0.045 to 11.0) greater reduction at 48 weeks. The numbers of participants at these time points were small (see Figure 1).

ANCOVA of MADRS and QIDS-C scale scores at 12 weeks, adjusting for baseline, found no significant difference between treatment arms (2.59 points, 95% CI: −4.54 to 9.72, *d* = 0.65; 2.30 points, 95% CI: −1.72 to 6.32, *d* = 0.34, respectively). QIDS-SR response and remission rates at 12 weeks were not significantly different (response 25% and 15%; remission 12.5% and 15% for pramipexole and placebo, respectively). However, at the point of exit from the study, there were significantly higher response and remission rates for pramipexole (*n* = 13) versus placebo (*n* = 16; response: 46% versus 6%, *p* = 0.026; remission: 31% versus 0%, *p* = 0.03 Fischer’s Exact test).

Pramipexole can be activated (Fawcett et al., 2016). However, there was no suggestion that pramipexole worsened anxiety (Supplemental Figure S2). ANCOVA of the GAD-7 data found a small difference of 1.32 points (95% CI: −0.87 to 3.50) between treatment arms at 12 weeks, but a larger difference at 36 weeks of 3.44 points (95% CI: −1.06 to 6.99) in favour of pramipexole.

SHAPS ratings of pleasure were 2.04 points lower in the pramipexole arm at week 6 (95% CI: −0.11 to 4.20), but only 0.76 points at week 12 (95% CI: −1.78 to 3.29) (Supplemental Figure S3).

**Figure 3.**
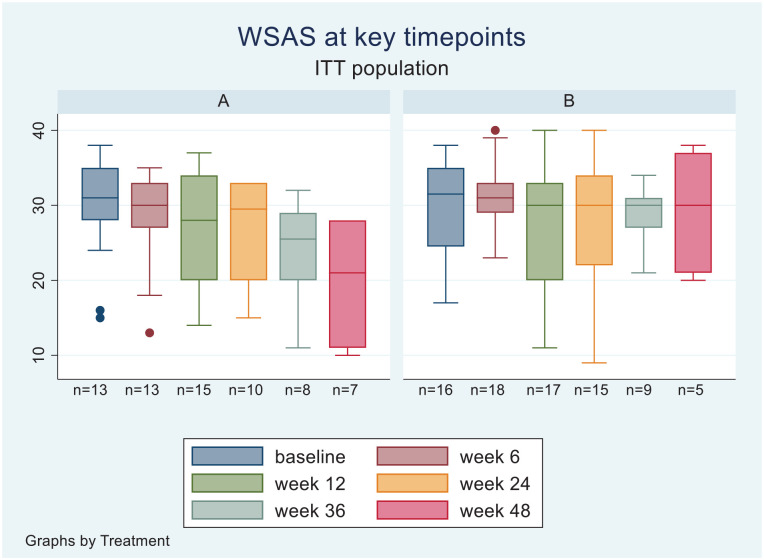
Boxplots of WSAS scores for completer populations at baseline, 6, 12, 24, 36 and 48 weeks.

ANOVA of WSAS assessment of psychosocial function, adjusting for baseline scores, revealed an advantage of pramipexole over placebo at weeks 36 and 48 with 5.36 (95% CI: 0.38–10.34, *d* = 0.80) and 9.63 (95% CI: 0.25–19.02, *d* = 0.98) point higher scores adjusting for baseline, but there was no difference at week 6, 12 or 24 (Figure 3).

### Adverse effects and tolerability

In terms of overall acceptability of treatment, TSQM scores were generally higher in the pramipexole arm (indicating better tolerability and acceptance of the treatment; Supplemental Figure S4). As can be seen from Figure 1, of the 36 participants reaching the 12-week primary outcome time point, 29 continued in the trial until either 48 weeks or when the study was terminated. There were two withdrawals of participants, one lost to follow-up and one with data not available in the pramipexole treatment arm and three withdrawals in the placebo arm. A further 14 participants were required to withdraw prior to 48 weeks due to the early closure of the trial.

Two hundred ninety AEs in the randomisation stage were reported by 36 of the 39 participants randomised: 128 reported by 17 of 18 unique participants in the pramipexole arm and 162 by 19 of 21 in the placebo arm. 265 (91%) required no action, 22 (8%) led to treatment interrupted or the dose reduced and 3 (1%) to study medication withdrawn. As expected, the most common categories of AEs were those related to psychiatric, gastrointestinal (particularly nausea) and nervous systems, with more moderately severe AEs in the pramipexole arm related to psychiatric and gastrointestinal symptoms. MEDRA categories of AEs occurring during the randomisation stage are reported in Supplemental Table S7.

There was one SAE (suicide by drug overdose) that occurred in the pre-randomisation stage. In the randomisation stage, five SAEs were reported by five individual participants. Three of these were by participants in the placebo arm (severe depressive episode with suicidality; foot surgery with overnight stay in hospital; death, likely by suicide). The first two were judged as unrelated to the study medication and the third was judged as being unable to determine relatedness. In the pramipexole arm, two SAEs were reported. One (colitis) was judged as being unrelated, but the other (‘manic relapse with psychotic symptoms leading to hospitalisation’) was judged as being related to the study medication.

At 12 weeks, ASRM self-rated score of manic symptoms was significantly higher in the pramipexole group by 1.2 points adjusting for baseline differences (95% CI: 0.02–2.42) at week 12 (Supplemental Figures S1 and S5). At all later time points, the difference between treatment arms was less than 1 point and not statistically significant. There were no differences in observer-rated YMRS scores at week 12, or the proportion of time being free of manic symptoms (defined as ASRM ⩽ 5 on weekly ratings: 88% vs 96% for pramipexole and placebo, respectively). In addition to the one SAE related to mania, AEs reported by participants relating to hypomanic/manic symptoms were more common for participants in the pramipexole arm (11 AEs reported by 8 out of 18 participants in the ITT sample (44%)) compared with the placebo arm (12 AEs reported by 6 out of 21 participants (29%)). The co-administration of an antipsychotic may have impacted the occurrence of hypomanic/manic symptoms which is described in more detail below.

ANCOVA did not reveal any differences in QUIP-RS impulsivity scores between treatment arms (Supplemental Figure S6). There were 11 AEs related to impulse control behaviours (gambling, internet gaming, buying, eating, hair picking) reported by six participants in the pramipexole arm (33%) compared with and eight from four participants in the placebo arm (19%), hinting at a possibly higher rate with the active drug.

### Impact of co-treatment with an antipsychotic

Of the 16 participants from the pramipexole arm included in the analysis, 9 were taking an antipsychotic at randomisation. Supplemental Figure S7 shows box plots of QIDS-SR and ASRM scores. Little difference is seen between the two groups of participants. The lack of randomisation between these sub-groups and the small numbers precluded further statistical analysis. It is perhaps noteworthy that of the 18 participants who experienced one or more AEs related to hypomania or mania, only three of these were taking an antipsychotic. Of these, two were on relatively low doses (risperidone 1mg and quetiapine 100 mg).

## Discussion

While the primary outcome of the PAX-BD study did not show a statistically significant effect of pramipexole compared to placebo on mood, as measured by the QIDS-SR at 12 weeks, in patients with TRBD there are still indications that pramipexole may have efficacy in bipolar depression. At the primary outcome time point of 12 weeks in PAX-BD, the reduction in QIDS-SR score in the pramipexole arm was double that seen in the placebo group and the effect size was medium to large (*d* = 0.72). The reduction of over four QIDS-SR points in the pramipexole arm would also be judged as being a clinically meaningful reduction (Jha et al., 2019; McIntyre et al., 2021). This suggests that the lack of a statistically significant (*p* = 0.085) effect on the primary outcome potentially reflects the small sample size (*n* = 36) due to the trial being stopped early. This is further supported by secondary and post hoc analyses revealing a statistically significantly greater reduction in QIDS-SR score after 36 weeks of treatment and significantly greater rates of response (46% vs 6%) and remission (31% vs 0%) with pramipexole at the point participants exited from the trial. Similarly, there were statistically significant improvements in work and social function with pramipexole at 36 and 48 weeks. Such observations are in line with clinical experience (Fawcett et al., 2016).

Two previous small RCTs (*n* = 22 and 21) of pramipexole in patients with bipolar depression were both positive at the primary outcome time point of 6 weeks (Goldberg et al., 2004; Zarate et al., 2004). This is not consistent with the current study given the lack of a significant effect on mood seen at 12 weeks, but which became evident at later time points. However, it may be consistent with a near significant improvement in the ability to experience pleasure seen at 6 weeks (see below). Either way, the data from the current study do suggest that future research needs to consider examining the effects of pramipexole over a longer period than just 6 weeks.

While the overall tolerability and acceptability of pramipexole were good, its use is not without challenges. High rates of impulse control problems have been reported in patients with Parkinson’s Disease treated with pramipexole and other dopamine agonists (Rizos et al., 2016). While there were no significant differences in ratings of impulsivity between the two treatment arms, 33% of participants in the pramipexole arm reported an AE related to such problems, compared with 19% in the placebo arm. Interpretation of this data is complicated by the association between impulsivity with BD per se (Chan et al., 2023) and the overlap between such problems and hypomania/mania.

However, the biggest issue in the PAX-BD trial was the occurrence of hypomanic and manic symptoms which resulted in the only medication-related SAE and AEs in 8 of the 16 participants in the pramipexole arm compared with 6 out of 20 in the placebo arm. While there was no difference in YMRS scores between treatment arms at 12 weeks, there was a significant elevation in self-rated ASRM scores. This increase in hypomanic/manic symptoms occurred early with the rate being significantly higher at 12 weeks in the pramipexole arm. Coincident with this early effect of pramipexole, there was a nearly significant improvement in the ability to experience pleasure seen at 6 weeks (an increase of 2.04 points on the SHAPS: 95% CI: −0.11 to 4.20, *d* = −0.76), though not at 12 weeks. Whether this is an early pointer to a possible mechanism of action of pramipexole in improving mood, or whether the result may have been confounded by early increases in hypomanic symptoms or participants being unblinded to treatment allocation as a result of side effects is unclear.

The use of an antipsychotic in combination with pramipexole is contentious. While it has been reported that the combination can be used successfully to treat BD in clinical practice (El-Mallakh et al., 2010), the funders for PAX-BD initially required participants to not be taking an antipsychotic. This, most probably, was due to the view that it was illogical to combine pramipexole (a dopamine agonist) with an antipsychotic (a dopamine antagonist). However, this takes a simplistic view of the drugs’ pharmacology. Pramipexole is a relatively ‘clean’ dopamine agonist, but it has activity at three dopamine receptors: D2, D3, and D4. The binding affinity of pramipexole is highest at the D3 receptor (Ki = 0.5 nM) – around an order of magnitude greater than at D2 (Ki = 3.9 nM) and D4 (Ki = 5.1 nM) receptors (Deutschländer et al., 2016; Kvernmo et al., 2006; Mierau et al., 1995). The pharmacology of antipsychotics varies, but in general, they have a higher affinity for D2 receptors compared with D3 receptors (Li et al., 2016; Richtand et al., 2007). Genetically modified mice that do not express D3 receptors have been reported to exhibit depressive and anxious features (Moraga-Amaro et al., 2014). In addition, a wealth of data using highly selective D3 antagonists in animal models has demonstrated that D3 receptors play a critical role in reward processes (Groman et al., 2020; Jordan et al., 2019) and PET imaging data in humans suggests D3 receptor expression may be related to motivation for rewards (Caravaggio et al., 2018). Such findings support a hypothesis that the mechanism of action of pramipexole in BD may be mediated via D3 receptors. On this basis, and with the high rate of use of antipsychotics in BD in clinical practice hampering recruitment, the PAX-BD protocol was amended to allow participants to be randomised while still taking an antipsychotic. Unfortunately, the relatively small numbers of participants in the pramipexole arm who were and were not taking an antipsychotic prevented statistical analysis. However, it appears that co-administration had little impact on the positive effects on depressive symptoms, but probably reduced the risk of manic symptoms. It is noteworthy that the one participant experiencing an SAE and seven who had an AE, related to mania while on pramipexole were not taking an antipsychotic. Conversely, only one participant on an antipsychotic experienced a hypomanic AE when taking pramipexole. Further research is required to clarify the impact of co-administration of an antipsychotic with pramipexole. Consideration of the relative affinity of different antipsychotics and pramipexole for D2 and D3 receptors may need to be taken into consideration when studying this. A starting point may be the range and dosages of antipsychotics described in Supplemental Table S3, the caution being that this is based upon the assumption that pramipexole’s beneficial effects in TRBD arise from its action on D3 receptors and that these doses have been derived entirely theoretically.

The uncertainties regarding the efficacy and safety of pramipexole for TRBD need to be addressed by a further, larger, study. PAX-BD has demonstrated that it is possible to conduct such a study, including with remote collection of data. The termination of the study before the intended sample size was reached was due to slow recruitment. This was heavily influenced by the impact of the COVID-19 pandemic. Following the pause in recruitment due to the COVID lockdowns, it took a considerable amount of time for the study to regain recruitment momentum. This, at least partially, reflected COVID-related clinical pressures and changes in practice (e.g. remote rather than face-to-face review of patients). The intended sample size may have also been somewhat overly ambitious. However, the study helps provide data to support sample size calculations for a future study. After the power calculation described in the methods section above, data that were not available at the time suggests that a more appropriate minimal clinical important difference (MCID) on the QIDS-SR is 4, rather than 3, points (Jha et al., 2019; McIntyre et al., 2021). A power calculation using the standard deviation for QIDS-SR derived from the PAX-BD data of 5 and an MCID of 4 suggests that 34 participants with TRBD would be required in each arm (68 in total) to achieve a 90% chance of detecting a clinically significant effect of pramipexole on mood. In the PAX-BD study, there were 12 dropouts in the pre-randomisation stage, of whom three were due to having to discontinue an antipsychotic. Excluding these three, this is a drop-out rate of 18%. Between randomisation and the primary outcome time point, there three participants dropped out (8%). Using estimates of 20% and 10% for drop-out rates in the pre- and randomisation stages, the total sample size required to successfully compare pramipexole with placebo in TRBD would be 95 recruited to the pre-randomisation stage in a future study using a similar design as PAX-BD. This is a much more pragmatic and feasible target compared with the sample size originally calculated for the PAX-BD study. It is possible that if the original target had been to recruit 95 rather than 414 participants, the funder may not have decided to prematurely close the study. The ethical approval for the PAX-BD study confined recruitment to those patients currently in secondary care. The Mood Disorders workstream of the UK Mental Health Mission (https://www.gov.uk/government/publications/life-sciences-vision-missions/mental-health-mission) is supporting the development of a network of research clinics to facilitate self and primary care referral into studies. Developments such as this are expected to facilitate recruitment for future clinical trials in mood disorders. A final point regarding future studies is that the PAX-BD demonstrated the importance of allowing the recruitment of participants both on, and not on, antipsychotics. The funder’s initial requirement that all participants must not be taking antipsychotics was a major impediment to recruitment. The study PPI group saw this requirement as a huge barrier to recruitment and advocated that it should be amended. In addition, the lack of tolerating withdrawal of antipsychotics in the pre-randomisation phase was a reason for not progressing to randomisation in a quarter of those who did not. While very tentative due to the small numbers, the study seems to demonstrate that response to pramipexole may well be possible in those still on antipsychotics, and hence the initial requirement to be antipsychotic-free would not be needed in a future study.

### Limitations

There are a number of issues with the PAX-BD study which limit interpretation of the data. Most notable is the small sample size with only 36 participants contributing primary outcome data. In addition, not all participants were able to continue follow-up to 48 weeks due to the early study closure. This resulted in particularly small sample sizes at the 36- and 48-week assessment time points, and participants exiting the study with variable follow-up periods of 16–48 weeks. As a result, significant effects seen at later follow-up time points could be due to attrition bias. The small sample size also hampers comparison of those on and not on an antipsychotic in combination with pramipexole. The small sample size is likely to have also contributed to the baseline differences in QIDS-SR scores. While analysis adjusted for this, there remains a concern that it may have biased the results.

The study was designed to investigate the efficacy of pramipexole in TRBD. There is little consensus as to how TRBD is defined but the available definitions focus on lack of response (Hidalgo-Mazzei et al., 2019). However, in clinical practice, a lack of tolerability or acceptability is an additional challenge. PAX-BD took a pragmatic, clinical, approach to defining TRBD, including lack of response, tolerability or acceptability. This does mean that 24% of participants randomised in PAX-BD had not failed to respond to even one of the four recommended drugs for bipolar depression in the current episode, and 41% had failed to respond to just one.

Additional limitations include the lack of an objective measure of adherence to study medication (precluded by the general remote conduct of the study) or check on study blinding, such as asking participants to state which treatment arm they believed they were in. Un-blinding may have occurred due to the side effect profile of pramipexole, though the rate of adverse events was similar between treatment arms. Assessments of AEs were often conducted remotely, which may have influenced the accuracy of their categorisation, for example, the ability to differentiate between the effects of long COVID and bipolar depressive symptoms. The requirement for online collection of outcome data may have biased participant selection. It should be noted that qualitative data (collected in the course of the PAX-BD study and published elsewhere) strongly supported the generally remote conduct of the study enabled by remote data collection (McAllister-Williams et al. Health Technology Assessment, NIHR Journals Library, in press).

## Conclusions

The PAX-BD study provides some further evidence that pramipexole might be an effective treatment for TRBD but unfortunately, the results are inconclusive given a clinically important effect that is not statistically significant at the primary outcome point. This was likely due to the early closure of the study. The longer-term follow-up in the study compared to previous RCTs suggests that the benefits of pramipexole may increase over time (supported by parallel ongoing economic evaluation of data from the study). It also provides valuable additional safety data. This does demonstrate an increase in subjective hypomanic/manic symptoms especially early in treatment, which may possibly be reduced by co-administration with an antipsychotic. An increase in impulsive symptoms was also seen. Further research in larger RCTs is required with the PAX-BD study providing data to guide study design and sample size needed.

## Supplemental Material

sj-docx-1-jop-10.1177_02698811241309622 – Supplemental material for A randomised double-blind, placebo-controlled trial of pramipexole in addition to mood stabilisers for patients with treatment-resistant bipolar depression (the PAX-BD study) Supplemental material, sj-docx-1-jop-10.1177_02698811241309622 for A randomised double-blind, placebo-controlled trial of pramipexole in addition to mood stabilisers for patients with treatment-resistant bipolar depression (the PAX-BD study) by R Hamish McAllister-Williams, Nicola Goudie, Lumbini Azim, Victoria Bartle, Michael Berger, Chrissie Butcher, Thomas Chadwick, Emily Clare, Paul Courtney, Lyndsey Dixon, Nichola Duffelen, Tony Fouweather, William Gann, John Geddes, Sumeet Gupta, Beth Hall, Timea Helter, Paul Hindmarch, Eva-Maria Holstein, Ward Lawrence, Phil Mawson, Iain McKinnon, Adam Milne, Aisling Molloy, Abigail Moore, Richard Morriss, Anisha Nakulan, Judit Simon, Daniel Smith, Bryony Stokes-Crossley, Paul RA Stokes, Andrew Swain, Adeola Taiwo, Zoë Walmsley, Christopher Weetman, Allan H Young and Stuart Watson in Journal of Psychopharmacology

sj-docx-10-jop-10.1177_02698811241309622 – Supplemental material for A randomised double-blind, placebo-controlled trial of pramipexole in addition to mood stabilisers for patients with treatment-resistant bipolar depression (the PAX-BD study) Supplemental material, sj-docx-10-jop-10.1177_02698811241309622 for A randomised double-blind, placebo-controlled trial of pramipexole in addition to mood stabilisers for patients with treatment-resistant bipolar depression (the PAX-BD study) by R Hamish McAllister-Williams, Nicola Goudie, Lumbini Azim, Victoria Bartle, Michael Berger, Chrissie Butcher, Thomas Chadwick, Emily Clare, Paul Courtney, Lyndsey Dixon, Nichola Duffelen, Tony Fouweather, William Gann, John Geddes, Sumeet Gupta, Beth Hall, Timea Helter, Paul Hindmarch, Eva-Maria Holstein, Ward Lawrence, Phil Mawson, Iain McKinnon, Adam Milne, Aisling Molloy, Abigail Moore, Richard Morriss, Anisha Nakulan, Judit Simon, Daniel Smith, Bryony Stokes-Crossley, Paul RA Stokes, Andrew Swain, Adeola Taiwo, Zoë Walmsley, Christopher Weetman, Allan H Young and Stuart Watson in Journal of Psychopharmacology

sj-docx-11-jop-10.1177_02698811241309622 – Supplemental material for A randomised double-blind, placebo-controlled trial of pramipexole in addition to mood stabilisers for patients with treatment-resistant bipolar depression (the PAX-BD study) Supplemental material, sj-docx-11-jop-10.1177_02698811241309622 for A randomised double-blind, placebo-controlled trial of pramipexole in addition to mood stabilisers for patients with treatment-resistant bipolar depression (the PAX-BD study) by R Hamish McAllister-Williams, Nicola Goudie, Lumbini Azim, Victoria Bartle, Michael Berger, Chrissie Butcher, Thomas Chadwick, Emily Clare, Paul Courtney, Lyndsey Dixon, Nichola Duffelen, Tony Fouweather, William Gann, John Geddes, Sumeet Gupta, Beth Hall, Timea Helter, Paul Hindmarch, Eva-Maria Holstein, Ward Lawrence, Phil Mawson, Iain McKinnon, Adam Milne, Aisling Molloy, Abigail Moore, Richard Morriss, Anisha Nakulan, Judit Simon, Daniel Smith, Bryony Stokes-Crossley, Paul RA Stokes, Andrew Swain, Adeola Taiwo, Zoë Walmsley, Christopher Weetman, Allan H Young and Stuart Watson in Journal of Psychopharmacology

sj-docx-12-jop-10.1177_02698811241309622 – Supplemental material for A randomised double-blind, placebo-controlled trial of pramipexole in addition to mood stabilisers for patients with treatment-resistant bipolar depression (the PAX-BD study) Supplemental material, sj-docx-12-jop-10.1177_02698811241309622 for A randomised double-blind, placebo-controlled trial of pramipexole in addition to mood stabilisers for patients with treatment-resistant bipolar depression (the PAX-BD study) by R Hamish McAllister-Williams, Nicola Goudie, Lumbini Azim, Victoria Bartle, Michael Berger, Chrissie Butcher, Thomas Chadwick, Emily Clare, Paul Courtney, Lyndsey Dixon, Nichola Duffelen, Tony Fouweather, William Gann, John Geddes, Sumeet Gupta, Beth Hall, Timea Helter, Paul Hindmarch, Eva-Maria Holstein, Ward Lawrence, Phil Mawson, Iain McKinnon, Adam Milne, Aisling Molloy, Abigail Moore, Richard Morriss, Anisha Nakulan, Judit Simon, Daniel Smith, Bryony Stokes-Crossley, Paul RA Stokes, Andrew Swain, Adeola Taiwo, Zoë Walmsley, Christopher Weetman, Allan H Young and Stuart Watson in Journal of Psychopharmacology

sj-docx-13-jop-10.1177_02698811241309622 – Supplemental material for A randomised double-blind, placebo-controlled trial of pramipexole in addition to mood stabilisers for patients with treatment-resistant bipolar depression (the PAX-BD study) Supplemental material, sj-docx-13-jop-10.1177_02698811241309622 for A randomised double-blind, placebo-controlled trial of pramipexole in addition to mood stabilisers for patients with treatment-resistant bipolar depression (the PAX-BD study) by R Hamish McAllister-Williams, Nicola Goudie, Lumbini Azim, Victoria Bartle, Michael Berger, Chrissie Butcher, Thomas Chadwick, Emily Clare, Paul Courtney, Lyndsey Dixon, Nichola Duffelen, Tony Fouweather, William Gann, John Geddes, Sumeet Gupta, Beth Hall, Timea Helter, Paul Hindmarch, Eva-Maria Holstein, Ward Lawrence, Phil Mawson, Iain McKinnon, Adam Milne, Aisling Molloy, Abigail Moore, Richard Morriss, Anisha Nakulan, Judit Simon, Daniel Smith, Bryony Stokes-Crossley, Paul RA Stokes, Andrew Swain, Adeola Taiwo, Zoë Walmsley, Christopher Weetman, Allan H Young and Stuart Watson in Journal of Psychopharmacology

sj-docx-14-jop-10.1177_02698811241309622 – Supplemental material for A randomised double-blind, placebo-controlled trial of pramipexole in addition to mood stabilisers for patients with treatment-resistant bipolar depression (the PAX-BD study) Supplemental material, sj-docx-14-jop-10.1177_02698811241309622 for A randomised double-blind, placebo-controlled trial of pramipexole in addition to mood stabilisers for patients with treatment-resistant bipolar depression (the PAX-BD study) by R Hamish McAllister-Williams, Nicola Goudie, Lumbini Azim, Victoria Bartle, Michael Berger, Chrissie Butcher, Thomas Chadwick, Emily Clare, Paul Courtney, Lyndsey Dixon, Nichola Duffelen, Tony Fouweather, William Gann, John Geddes, Sumeet Gupta, Beth Hall, Timea Helter, Paul Hindmarch, Eva-Maria Holstein, Ward Lawrence, Phil Mawson, Iain McKinnon, Adam Milne, Aisling Molloy, Abigail Moore, Richard Morriss, Anisha Nakulan, Judit Simon, Daniel Smith, Bryony Stokes-Crossley, Paul RA Stokes, Andrew Swain, Adeola Taiwo, Zoë Walmsley, Christopher Weetman, Allan H Young and Stuart Watson in Journal of Psychopharmacology

sj-docx-2-jop-10.1177_02698811241309622 – Supplemental material for A randomised double-blind, placebo-controlled trial of pramipexole in addition to mood stabilisers for patients with treatment-resistant bipolar depression (the PAX-BD study) Supplemental material, sj-docx-2-jop-10.1177_02698811241309622 for A randomised double-blind, placebo-controlled trial of pramipexole in addition to mood stabilisers for patients with treatment-resistant bipolar depression (the PAX-BD study) by R Hamish McAllister-Williams, Nicola Goudie, Lumbini Azim, Victoria Bartle, Michael Berger, Chrissie Butcher, Thomas Chadwick, Emily Clare, Paul Courtney, Lyndsey Dixon, Nichola Duffelen, Tony Fouweather, William Gann, John Geddes, Sumeet Gupta, Beth Hall, Timea Helter, Paul Hindmarch, Eva-Maria Holstein, Ward Lawrence, Phil Mawson, Iain McKinnon, Adam Milne, Aisling Molloy, Abigail Moore, Richard Morriss, Anisha Nakulan, Judit Simon, Daniel Smith, Bryony Stokes-Crossley, Paul RA Stokes, Andrew Swain, Adeola Taiwo, Zoë Walmsley, Christopher Weetman, Allan H Young and Stuart Watson in Journal of Psychopharmacology

sj-docx-3-jop-10.1177_02698811241309622 – Supplemental material for A randomised double-blind, placebo-controlled trial of pramipexole in addition to mood stabilisers for patients with treatment-resistant bipolar depression (the PAX-BD study) Supplemental material, sj-docx-3-jop-10.1177_02698811241309622 for A randomised double-blind, placebo-controlled trial of pramipexole in addition to mood stabilisers for patients with treatment-resistant bipolar depression (the PAX-BD study) by R Hamish McAllister-Williams, Nicola Goudie, Lumbini Azim, Victoria Bartle, Michael Berger, Chrissie Butcher, Thomas Chadwick, Emily Clare, Paul Courtney, Lyndsey Dixon, Nichola Duffelen, Tony Fouweather, William Gann, John Geddes, Sumeet Gupta, Beth Hall, Timea Helter, Paul Hindmarch, Eva-Maria Holstein, Ward Lawrence, Phil Mawson, Iain McKinnon, Adam Milne, Aisling Molloy, Abigail Moore, Richard Morriss, Anisha Nakulan, Judit Simon, Daniel Smith, Bryony Stokes-Crossley, Paul RA Stokes, Andrew Swain, Adeola Taiwo, Zoë Walmsley, Christopher Weetman, Allan H Young and Stuart Watson in Journal of Psychopharmacology

sj-docx-4-jop-10.1177_02698811241309622 – Supplemental material for A randomised double-blind, placebo-controlled trial of pramipexole in addition to mood stabilisers for patients with treatment-resistant bipolar depression (the PAX-BD study) Supplemental material, sj-docx-4-jop-10.1177_02698811241309622 for A randomised double-blind, placebo-controlled trial of pramipexole in addition to mood stabilisers for patients with treatment-resistant bipolar depression (the PAX-BD study) by R Hamish McAllister-Williams, Nicola Goudie, Lumbini Azim, Victoria Bartle, Michael Berger, Chrissie Butcher, Thomas Chadwick, Emily Clare, Paul Courtney, Lyndsey Dixon, Nichola Duffelen, Tony Fouweather, William Gann, John Geddes, Sumeet Gupta, Beth Hall, Timea Helter, Paul Hindmarch, Eva-Maria Holstein, Ward Lawrence, Phil Mawson, Iain McKinnon, Adam Milne, Aisling Molloy, Abigail Moore, Richard Morriss, Anisha Nakulan, Judit Simon, Daniel Smith, Bryony Stokes-Crossley, Paul RA Stokes, Andrew Swain, Adeola Taiwo, Zoë Walmsley, Christopher Weetman, Allan H Young and Stuart Watson in Journal of Psychopharmacology

sj-docx-5-jop-10.1177_02698811241309622 – Supplemental material for A randomised double-blind, placebo-controlled trial of pramipexole in addition to mood stabilisers for patients with treatment-resistant bipolar depression (the PAX-BD study) Supplemental material, sj-docx-5-jop-10.1177_02698811241309622 for A randomised double-blind, placebo-controlled trial of pramipexole in addition to mood stabilisers for patients with treatment-resistant bipolar depression (the PAX-BD study) by R Hamish McAllister-Williams, Nicola Goudie, Lumbini Azim, Victoria Bartle, Michael Berger, Chrissie Butcher, Thomas Chadwick, Emily Clare, Paul Courtney, Lyndsey Dixon, Nichola Duffelen, Tony Fouweather, William Gann, John Geddes, Sumeet Gupta, Beth Hall, Timea Helter, Paul Hindmarch, Eva-Maria Holstein, Ward Lawrence, Phil Mawson, Iain McKinnon, Adam Milne, Aisling Molloy, Abigail Moore, Richard Morriss, Anisha Nakulan, Judit Simon, Daniel Smith, Bryony Stokes-Crossley, Paul RA Stokes, Andrew Swain, Adeola Taiwo, Zoë Walmsley, Christopher Weetman, Allan H Young and Stuart Watson in Journal of Psychopharmacology

sj-docx-6-jop-10.1177_02698811241309622 – Supplemental material for A randomised double-blind, placebo-controlled trial of pramipexole in addition to mood stabilisers for patients with treatment-resistant bipolar depression (the PAX-BD study) Supplemental material, sj-docx-6-jop-10.1177_02698811241309622 for A randomised double-blind, placebo-controlled trial of pramipexole in addition to mood stabilisers for patients with treatment-resistant bipolar depression (the PAX-BD study) by R Hamish McAllister-Williams, Nicola Goudie, Lumbini Azim, Victoria Bartle, Michael Berger, Chrissie Butcher, Thomas Chadwick, Emily Clare, Paul Courtney, Lyndsey Dixon, Nichola Duffelen, Tony Fouweather, William Gann, John Geddes, Sumeet Gupta, Beth Hall, Timea Helter, Paul Hindmarch, Eva-Maria Holstein, Ward Lawrence, Phil Mawson, Iain McKinnon, Adam Milne, Aisling Molloy, Abigail Moore, Richard Morriss, Anisha Nakulan, Judit Simon, Daniel Smith, Bryony Stokes-Crossley, Paul RA Stokes, Andrew Swain, Adeola Taiwo, Zoë Walmsley, Christopher Weetman, Allan H Young and Stuart Watson in Journal of Psychopharmacology

sj-docx-7-jop-10.1177_02698811241309622 – Supplemental material for A randomised double-blind, placebo-controlled trial of pramipexole in addition to mood stabilisers for patients with treatment-resistant bipolar depression (the PAX-BD study) Supplemental material, sj-docx-7-jop-10.1177_02698811241309622 for A randomised double-blind, placebo-controlled trial of pramipexole in addition to mood stabilisers for patients with treatment-resistant bipolar depression (the PAX-BD study) by R Hamish McAllister-Williams, Nicola Goudie, Lumbini Azim, Victoria Bartle, Michael Berger, Chrissie Butcher, Thomas Chadwick, Emily Clare, Paul Courtney, Lyndsey Dixon, Nichola Duffelen, Tony Fouweather, William Gann, John Geddes, Sumeet Gupta, Beth Hall, Timea Helter, Paul Hindmarch, Eva-Maria Holstein, Ward Lawrence, Phil Mawson, Iain McKinnon, Adam Milne, Aisling Molloy, Abigail Moore, Richard Morriss, Anisha Nakulan, Judit Simon, Daniel Smith, Bryony Stokes-Crossley, Paul RA Stokes, Andrew Swain, Adeola Taiwo, Zoë Walmsley, Christopher Weetman, Allan H Young and Stuart Watson in Journal of Psychopharmacology

sj-docx-8-jop-10.1177_02698811241309622 – Supplemental material for A randomised double-blind, placebo-controlled trial of pramipexole in addition to mood stabilisers for patients with treatment-resistant bipolar depression (the PAX-BD study) Supplemental material, sj-docx-8-jop-10.1177_02698811241309622 for A randomised double-blind, placebo-controlled trial of pramipexole in addition to mood stabilisers for patients with treatment-resistant bipolar depression (the PAX-BD study) by R Hamish McAllister-Williams, Nicola Goudie, Lumbini Azim, Victoria Bartle, Michael Berger, Chrissie Butcher, Thomas Chadwick, Emily Clare, Paul Courtney, Lyndsey Dixon, Nichola Duffelen, Tony Fouweather, William Gann, John Geddes, Sumeet Gupta, Beth Hall, Timea Helter, Paul Hindmarch, Eva-Maria Holstein, Ward Lawrence, Phil Mawson, Iain McKinnon, Adam Milne, Aisling Molloy, Abigail Moore, Richard Morriss, Anisha Nakulan, Judit Simon, Daniel Smith, Bryony Stokes-Crossley, Paul RA Stokes, Andrew Swain, Adeola Taiwo, Zoë Walmsley, Christopher Weetman, Allan H Young and Stuart Watson in Journal of Psychopharmacology

sj-docx-9-jop-10.1177_02698811241309622 – Supplemental material for A randomised double-blind, placebo-controlled trial of pramipexole in addition to mood stabilisers for patients with treatment-resistant bipolar depression (the PAX-BD study) Supplemental material, sj-docx-9-jop-10.1177_02698811241309622 for A randomised double-blind, placebo-controlled trial of pramipexole in addition to mood stabilisers for patients with treatment-resistant bipolar depression (the PAX-BD study) by R Hamish McAllister-Williams, Nicola Goudie, Lumbini Azim, Victoria Bartle, Michael Berger, Chrissie Butcher, Thomas Chadwick, Emily Clare, Paul Courtney, Lyndsey Dixon, Nichola Duffelen, Tony Fouweather, William Gann, John Geddes, Sumeet Gupta, Beth Hall, Timea Helter, Paul Hindmarch, Eva-Maria Holstein, Ward Lawrence, Phil Mawson, Iain McKinnon, Adam Milne, Aisling Molloy, Abigail Moore, Richard Morriss, Anisha Nakulan, Judit Simon, Daniel Smith, Bryony Stokes-Crossley, Paul RA Stokes, Andrew Swain, Adeola Taiwo, Zoë Walmsley, Christopher Weetman, Allan H Young and Stuart Watson in Journal of Psychopharmacology
