# Supplementary figures and images for: A randomised double-blind, placebo-controlled trial of pramipexole in addition to mood stabilisers for patients with treatment-resistant bipolar depression (the PAX-BD study)
Source: J Psychopharmacol. 2025 Jan 20;39(2):106–20. doi: 10.1177/02698811241309622 (PMC11831867; doi:10.1177/02698811241309622)

Figure S1 - Weekly QIDS-SR and ASRM scores from randomisation to a maximum of 48 weeks


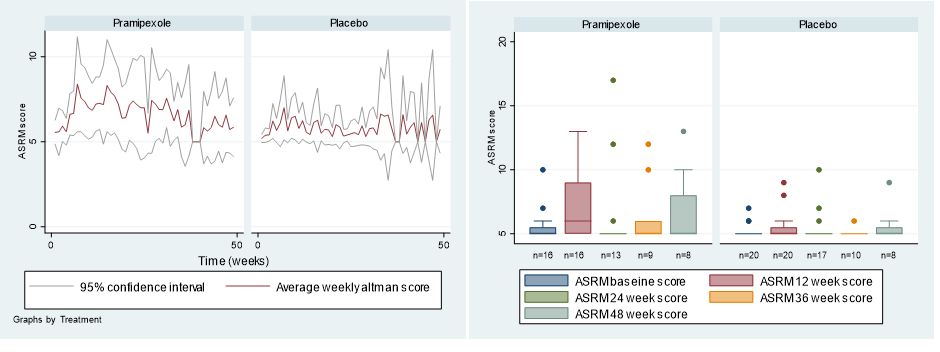

Supplement: sj-docx-1-jop-10.1177_02698811241309622 – Supplemental material for A randomised double-blind, placebo-controlled trial of pramipexole in addition to mood stabilisers for patients with treatment-resistant bipolar depression (the PAX-BD study) [file sj-docx-1-jop-10.1177_02698811241309622.docx]

**Figure S2** - Boxplots of GAD-7 scores for completer populations at baseline, 12, 24, 36 and 48 weeks


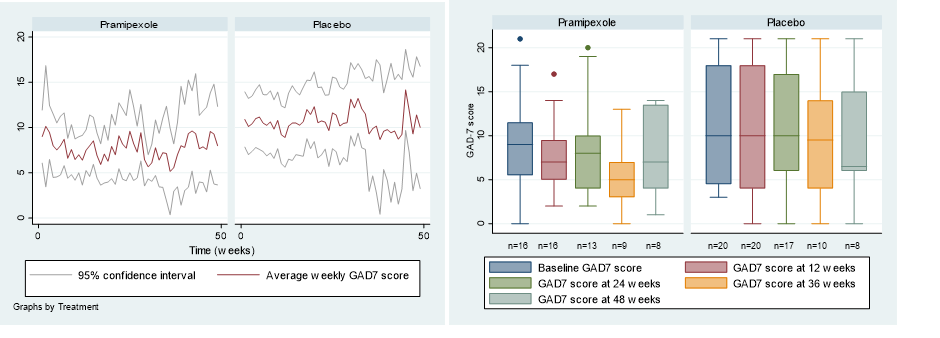

Supplement: sj-docx-2-jop-10.1177_02698811241309622 – Supplemental material for A randomised double-blind, placebo-controlled trial of pramipexole in addition to mood stabilisers for patients with treatment-resistant bipolar depression (the PAX-BD study) [file sj-docx-2-jop-10.1177_02698811241309622.docx]

Figure S3 - Boxplots of SHAPS scores for completer populations at baseline, 6 and 12 weeks


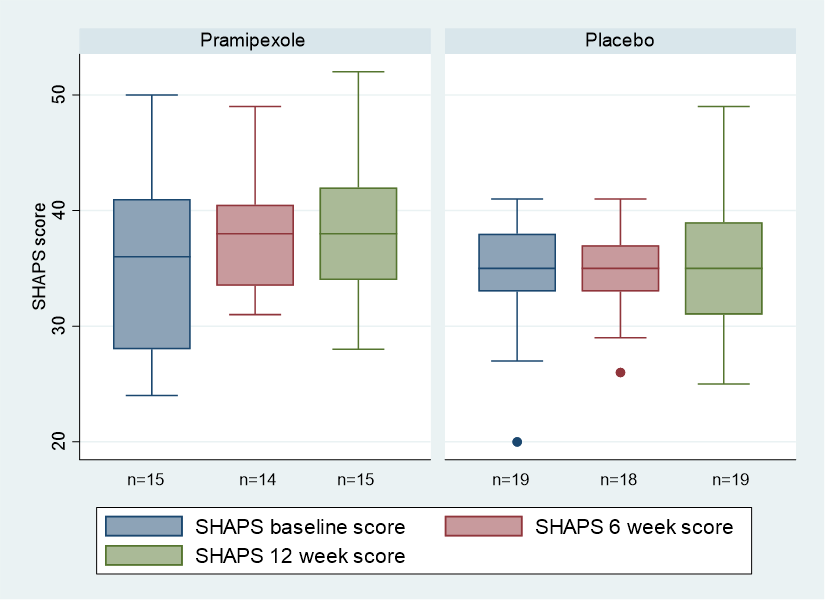

Supplement: sj-docx-3-jop-10.1177_02698811241309622 – Supplemental material for A randomised double-blind, placebo-controlled trial of pramipexole in addition to mood stabilisers for patients with treatment-resistant bipolar depression (the PAX-BD study) [file sj-docx-3-jop-10.1177_02698811241309622.docx]

Figure S4 - Boxplots of TSQM scores for completer populations at 12, 24, 36 and 48 weeks.


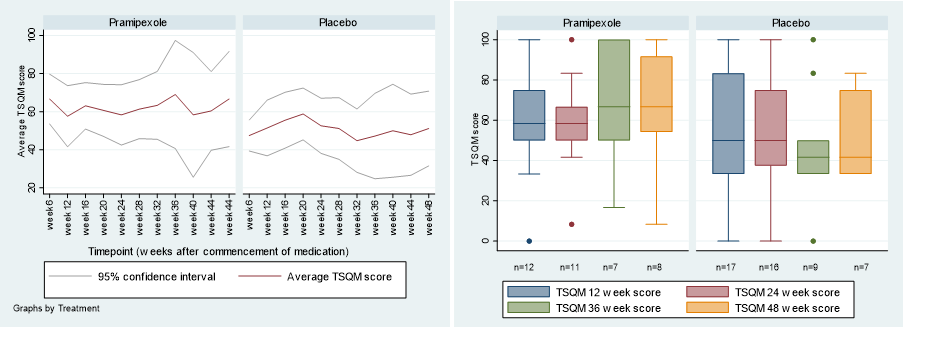

Supplement: sj-docx-4-jop-10.1177_02698811241309622 – Supplemental material for A randomised double-blind, placebo-controlled trial of pramipexole in addition to mood stabilisers for patients with treatment-resistant bipolar depression (the PAX-BD study) [file sj-docx-4-jop-10.1177_02698811241309622.docx]

Figure S5 - Boxplots of ASRM scores for completer populations at baseline, 12, 24, 36 and 48 weeks.


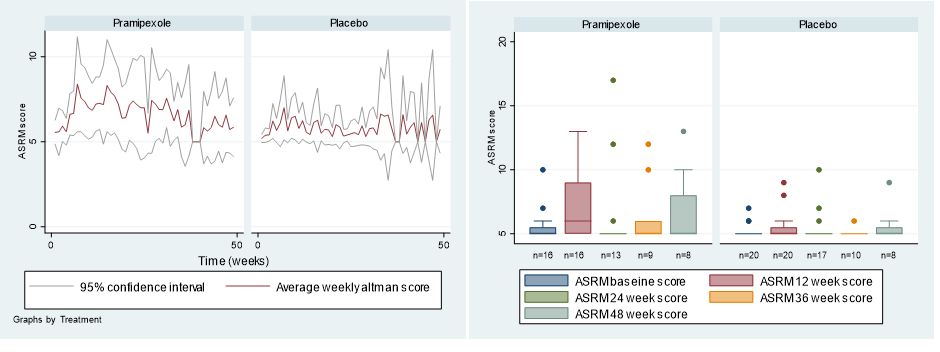

Supplement: sj-docx-5-jop-10.1177_02698811241309622 – Supplemental material for A randomised double-blind, placebo-controlled trial of pramipexole in addition to mood stabilisers for patients with treatment-resistant bipolar depression (the PAX-BD study) [file sj-docx-5-jop-10.1177_02698811241309622.docx]

Figure S6 - Boxplots of QUIP-RS scores for completer populations at baseline, 12, 24, 36 and 48 weeks.


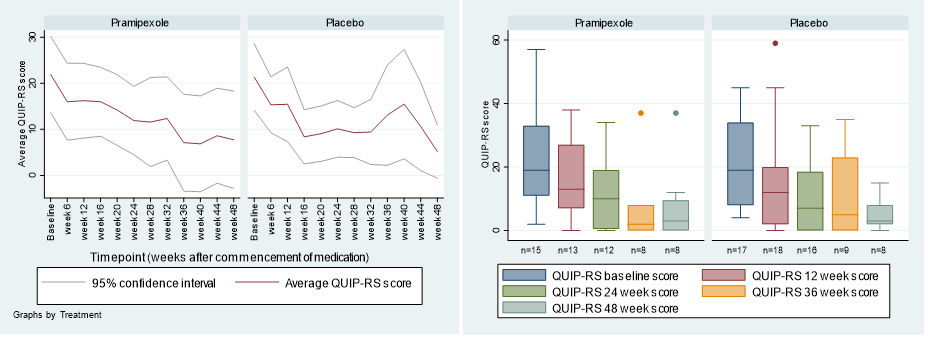

Supplement: sj-docx-6-jop-10.1177_02698811241309622 – Supplemental material for A randomised double-blind, placebo-controlled trial of pramipexole in addition to mood stabilisers for patients with treatment-resistant bipolar depression (the PAX-BD study) [file sj-docx-6-jop-10.1177_02698811241309622.docx]
