# Supplementary material for: A randomised double-blind, placebo-controlled trial of pramipexole in addition to mood stabilisers for patients with treatment-resistant bipolar depression (the PAX-BD study)
Source: J Psychopharmacol. 2025 Jan 20;39(2):106–20. doi: 10.1177/02698811241309622 (PMC11831867; doi:10.1177/02698811241309622)
Supplement: sj-docx-10-jop-10.1177_02698811241309622 – Supplemental material for A randomised double-blind, placebo-controlled trial of pramipexole in addition to mood stabilisers for patients with treatment-resistant bipolar depression (the PAX-BD study) [file sj-docx-10-jop-10.1177_02698811241309622.docx]

Table S3: Maximum daily dose of various antipsychotics allowed for eligibility to randomisation in PAX-BD following protocol amendment

| **Drug** | **Maximum daily dose allowed for eligibility to randomisation stage** |
| --- | --- |
| Aripiprazole | 15mg |
| Aripiprazole depot | 400mg every 4 weeks |
| Chlorpromazine | 200mg |
| Flupentixol depot | 200mg every 4 weeks |
| Haloperidol | 2mg |
| Haloperidol depot | 100mg every 4 weeks |
| Lurasidone | 111mg |
| Olanzapine | 10mg |
| Olanzapine depot | 150mg every 2 weeks |
| Paliperidone | 3mg |
| Paliperidone monthly depot | 75mg every month |
| Paliperidone 3 monthly depot | 263mg every 3 months |
| Quetiapine | 300mg |
| Risperidone | 1mg |
| Risperidone depot | 25mg every 2 weeks |
| Zuclopenthixol depot | 500mg every 4 weeks |
