# Supplementary material for: A randomised double-blind, placebo-controlled trial of pramipexole in addition to mood stabilisers for patients with treatment-resistant bipolar depression (the PAX-BD study)
Source: J Psychopharmacol. 2025 Jan 20;39(2):106–20. doi: 10.1177/02698811241309622 (PMC11831867; doi:10.1177/02698811241309622)
Supplement: sj-docx-11-jop-10.1177_02698811241309622 – Supplemental material for A randomised double-blind, placebo-controlled trial of pramipexole in addition to mood stabilisers for patients with treatment-resistant bipolar depression (the PAX-BD study) [file sj-docx-11-jop-10.1177_02698811241309622.docx]

Table S4. Medication history of participants in the various stages of the trial

|  | **Mean daily dose (range) in mg unless otherwise stated** | **Pre-randomisation:did not progress (N=12)** | **Pramipexole arm (n=18)** | **Placebo arm (n=21)** |
| --- | --- | --- | --- | --- |
| **Currently taking an antipsychotics**  Yes  No |  | 9 (75%)  3 (25%) | 10 (56%)  8 (44%) | 10 (48%)  11 (52%) |
| **Antipsychotics taken^1^**  Aripiprazole  Flupenthixol  Haloperidol  Lurasidone  Olanzapine  Paliperidone  Quetiapine  Risperidone | 18.8(10-30)  50(50)  2(2)  78.5(55.5-110)  11.8(2.5-20)  350(175-525)*****  281 (100-600)  1(1) | N=10  (9 unique)  2  0  1  1  3  1  2  0 | N=7  (7 unique)  1  1  0  1  1  0  3  0 | N=11  (10 unique)  1  0  0  2  2  1  5  0 |
| **Currently taking mood stabilisers**  Yes  No |  | 5 (42%)  7 (58%) | 17 (94%)  1 (6%) | 20 (95%)  1 (5%) |
| **Mood stabilisers taken^2^**  Lamotrigine  Lithium  Oxcarbamazepine  Valproate | 241(25-400)  760(400-1600)  600(600)  1060(500-1500) | N=5  (5 unique)  1  1  0  3 | 20  (17 unique)  10  7  0  3 | 26  (20 unique)  11  10  1  4 |
| **Currently taking anti-depressants**  Yes  No |  | 7 (58%)  5 (42%) | 11 (61%)  7 (49%) | 11 (52%)  10 (48%) |
| **Antidepressants taken^3^**  SSRIs   - Citalopram - Escitalopram - Fluoxetine - Paroxetine - Sertraline   SNRI   - Venlafaxine   TCAs   - Amitriptyline - Dosulepin - Lofepramine   Others   - Mirtazapine - Trazodone - Vortioxetine | 10(10)  3.8(2.5-5)  42.5(20-60)  25(10-50)  150(100-200)  225(75-375)  100(50150)  75(75)  210(210)  33(15-45)  50(50)  15(10-20) | N=8  (7 unique)  1  0  1  0  2  2  0  0  0  1  1  0 | N=12  (11 unique)  0  0  2  1  0  4  1  1  1  1  0  1 | N=15  (11 unique)  0  2  5  1  0  2  1  0  0  3  0  1 |
| **Anxiolytics/**  **Hypnotics**  Yes  No |  | 7 (58%)  5 (42%) | 8 (44%)  10 (56%) | 10 (48%)  11 (52%) |
| **Anxiolytics/**  **Hypnotics taken^4^**  Benzodiazepines   - Clonazepam - Diazepam - Lorazepam - Temazepam   Z drugs   - Zolpidem - Zopiclone   Others   - Buspirone - Gabapentin - Pregabalin - Melatonin - Promethazine | 2(2)  5.7(2-11)  1.4(1-2)  10(10)  5(5)  7.5(7.5)  15(15)  900(900)  367(150-600)  2(2)  25(25) | N=9  (7 unique)  0  1  2  0  0  3  0  0  2  0  1 | N=8  (8 unique)  0  1  1  1  0  2  1  1  1  0  0 | N=14  (10 unique)  1  4  2  0  1  1  0  0  3  1  1 |
| **Currently taking other psychotropics^5^**  Yes  No |  | 2 (17%)  10 (83%) | 4 (19%)  14 (81%) | 4 (19%)  17 (81%) |
| **Currently taking non-psychotropic medication**  Yes  No |  | 7 (58%)  5 (42%) | 13 (72%)  5 (28%) | 13 (62%)  8 (38%) |

**Note that statements regarding whether medication was taken relates to entry to the pre-randomisation stage. All participants were taking a mood stabiliser at the point of entry to the randomisation stage.**

*** -** monthly depot injection

** -

1 – One participant was on a combination of lurasidone + quetiapine and another on paliperidone depot injection + oral haloperidol.

2 – Four participants were taking lithium + lamotrigine, three lamotrigine + valproate; two lithium + valproate and one lithium + oxcarbazepine.

3 – Five participants were taking mirtazapine + another antidepressant: two on venlafaxine and one each on dosulepine, vortioxetine and fluoxetine.

4 – Two participants were on combinations of three anxiolytics/hypnotics.

5 – “other” psychotropics included two participants on modafinil and one each on methylphenidate, pregabalin, procyclidine and promethazine.
