# Supplementary material for: A randomised double-blind, placebo-controlled trial of pramipexole in addition to mood stabilisers for patients with treatment-resistant bipolar depression (the PAX-BD study)
Source: J Psychopharmacol. 2025 Jan 20;39(2):106–20. doi: 10.1177/02698811241309622 (PMC11831867; doi:10.1177/02698811241309622)
Supplement: sj-docx-12-jop-10.1177_02698811241309622 – Supplemental material for A randomised double-blind, placebo-controlled trial of pramipexole in addition to mood stabilisers for patients with treatment-resistant bipolar depression (the PAX-BD study) [file sj-docx-12-jop-10.1177_02698811241309622.docx]

Table S5. Assessment of criteria for TRBD: rates of positive responses to “inadequate response”, “intolerance” and “declined/clinically inappropriate

| **Medication** | **Inadequate response** | **Intolerance** | **Declined/ Clinically inappropriate** |
| --- | --- | --- | --- |
|  | | | |
| **Full pre-randomisation sample (n=51)** | | | |
| **Olanzapine** | 12 (25%) | 10 (20%) | 26 (51%) |
| **Quetiapine** | 20 (39%) | 16 (31%) | 26 (51% |
| **Lurasidone** | 7 (14%) | 3 (6%) | 13 (25%) |
| **Lamotrigine** | 21 (41%) | 5 (10%) | 14 (27%) |
|  | | | |
| **Randomised (n=39)** | | | |
| **Olanzapine** | 9 (23%) | 8 (21%) | 19 (49%) |
| **Quetiapine** | 16 (41%) | 14 (36%) | 19 (49%) |
| **Lurasidone** | 5 (13%) | 3 (8%) | 9 (23%) |
| **Lamotrigine** | 19 (49%) | 5 (13%) | 9 (23%) |
|  | | | |
| **Not randomised (n=12)** | | | |
| **Olanzapine** | 3 (25%) | 2 (17%) | 7 (58%) |
| **Quetiapine** | 4 (33%) | 2 (17%) | 7 (58%) |
| **Lurasidone** | 2 (17%) | 0 (0%) | 4 (33%) |
| **Lamotrigine** | 3 (25%) | 0 (0%) | 5 (42%) |

Note that these criteria were judged clinically with regards to the current episode of bipolar depression. If a medication had been trialled in a previous episode and either there had been a non-response or intolerance, this would be grounds for indicating it to be “clinically inappropriate” in the current episode. For an “inadequate response” there needed to have been a minimum of an 8 week trial of olanzapine at a minimum dose of 5mg/day, quetiapine 300mg/day or lurasidone 55.5mg/day, or a trial of lamotrigine at a minimum of 200mg for at least 12 weeks. If these doses or duration were not tolerated, this would be grounds for a positive response to “intolerance".
