# Supplementary material for: A randomised double-blind, placebo-controlled trial of pramipexole in addition to mood stabilisers for patients with treatment-resistant bipolar depression (the PAX-BD study)
Source: J Psychopharmacol. 2025 Jan 20;39(2):106–20. doi: 10.1177/02698811241309622 (PMC11831867; doi:10.1177/02698811241309622)
Supplement: sj-docx-13-jop-10.1177_02698811241309622 – Supplemental material for A randomised double-blind, placebo-controlled trial of pramipexole in addition to mood stabilisers for patients with treatment-resistant bipolar depression (the PAX-BD study) [file sj-docx-13-jop-10.1177_02698811241309622.docx]

Table S6. Rates of multiple “inadequate” responses when assessing TRBD criteria.

| **Number of inadequate responses** | **Full pre-randomisation sample (n=51)** | **Randomised (n=39)** | **Not randomised (n=12)** |
| --- | --- | --- | --- |
| 0 | 12 (24%) | 8 (21%) | 4 (33%) |
| 1 | 21 (41%) | 17 (44%) | 4 (33%) |
| 2 | 15 (29%) | 11 (28%) | 4 (33%) |
| 3 | 2 (4%) | 2 (5%) | 0 (0%) |
| 4 | 1 (2%) | 1 (3%) | 0 (0%) |
| **Total** | **51 (100%)** | **39 (100%)** | **12 (100%)** |
