# Supplementary material for: A randomised double-blind, placebo-controlled trial of pramipexole in addition to mood stabilisers for patients with treatment-resistant bipolar depression (the PAX-BD study)
Source: J Psychopharmacol. 2025 Jan 20;39(2):106–20. doi: 10.1177/02698811241309622 (PMC11831867; doi:10.1177/02698811241309622)
Supplement: sj-docx-14-jop-10.1177_02698811241309622 – Supplemental material for A randomised double-blind, placebo-controlled trial of pramipexole in addition to mood stabilisers for patients with treatment-resistant bipolar depression (the PAX-BD study) [file sj-docx-14-jop-10.1177_02698811241309622.docx]

Table S7: MEDRA categories of AEs occurring during the randomisation stage

| **MEDRA classifications** |  | **Pramipexole (n=48)** | | | **Placebo (n=43)** | | |
| --- | --- | --- | --- | --- | --- | --- | --- |
| **Definitely related to IMP** |  | **severe** | **moderate** | **mild** | **severe** | **moderate** | **mild** |
|  | Psychiatric disorders | 1 (2%) | 10 (21%) | 7 (15%) | 0 (0%) | 5 (12%) | 8 (19%) |
|  | Gastrointestinal disorders | 0 (0%) | 6 (13%) | 13 (27%) | 0 (0%) | 0 (0%) | 11 (26%) |
|  | Nervous system disorders | 0 (0%) | 2 (4%) | 2 (4%) | 0 (0%) | 4 (9%) | 6 (14%) |
|  | Injury, poisoning and procedural complications | 0 (0%) | 1 (2%) | 0 (0%) | 0 (0%) | 1 (2%) | 1 (2%) |
|  | General disorders and administration site conditions | 0 (0%) | 1 (2%) | 0 (0%) | 0 (0%) | 2 (5%) | 2 (5%) |
|  | Skin and subcutaneous tissue disorders | 0 (0%) | 1 (2%) | 3 (6%) | 0 (0%) | 0 (0%) | 0 (0%) |
|  | Social circumstances | 0 (0%) | 0 (0%) | 0 (0%) | 0 (0%) | 0 (0%) | 1 (2%) |
|  | Investigations | 0 (0%) | 0 (0%) | 1 (2%) | 0 (0%) | 0 (0%) | 0 (0%) |
|  | Vascular disorders | 0 (0%) | 0 (0%) | 0 (0%) | 0 (0%) | 0 (0%) | 1 (2%) |
|  | Metabolism and nutrition disorders | 0 (0%) | 0 (0%) | 0 (0%) | 0 (0%) | 0 (0%) | 1 (2%) |
|  |  | **Pramipexole (n=27)** | | | **Placebo (n=39)** | | |
|  |  | **severe** | **moderate** | **mild** | **severe** | **moderate** | **mild** |
| **Unable to determine if related** | Psychiatric disorders | 1 (4%) | 9 (33%) | 4 (15%) | 0 (0%) | 10 (27%) | 8 (22%) |
|  | Gastrointestinal disorders | 0 (0%) | 0 (0%) | 1 (4%) | 0 (0%) | 4 (11%) | 3 (8%) |
|  | Nervous system disorders | 0 (0%) | 1 (4%) | 4 (15%) | 2 (5%) | 1 (3%) | 3 (8%) |
|  | General disorders and administration site conditions | 0 (0%) | 2 (7%) | 0 (0%) | 1 (3%) | 0 (0%) | 2 (5%) |
|  | Skin and subcutaneous tissue disorders | 0 (0%) | 0 (0%) | 1 (4%) | 0 (0%) | 0 (0%) | 0 (0%) |
|  | Eye disorders | 0 (0%) | 0 (0%) | 3 (11%) | 0 (0%) | 0 (0%) | 0 (0%) |
|  | Vascular disorders | 0 (0%) | 0 (0%) | 0 (0%) | 0 (0%) | 0 (0%) | 2 (5%) |
|  | Metabolism and nutrition disorders | 0 (0%) | 1 (4%) | 0 (0%) | 0 (0%) | 0 (0%) | 0 (0%) |
|  | Ear and labyrinth disorders | 0 (0%) | 0 (0%) | 0 (0%) | 0 (0%) | 1 (3%) | 0 (0%) |
| **Unrelated to IMP** |  | **Pramipexole (n=53)** | | | **Placebo (n=82)** | | |
|  |  | severe | mod | mild | severe | mod | mild |
|  | Psychiatric disorders | 0 (0%) | 4 (8%) | 5 (9%) | 0 (0%) | 8 (10%) | 14 (17%) |
|  | Gastrointestinal disorders | 0 (0%) | 1 (2%) | 4 (8%) | 0 (0%) | 1 (1%) | 9 (11%) |
|  | Nervous system disorders | 0 (0%) | 0 (0%) | 8 (15%) | 0 (0%) | 1 (1%) | 5 (6%) |
|  | Infections and infestations | 0 (0%) | 4 (8%) | 10 (19%) | 1 (1%) | 4 (5%) | 9 (11%) |
|  | Injury, poisoning and procedural complications | 0 (0%) | 2 (4%) | 3 (6%) | 1 (1%) | 1 (1%) | 15 (18%) |
|  | General disorders and administration site conditions | 0 (0%) | 0 (0%) | 4 (8%) | 0 (0%) | 1 (1%) | 0 (0%) |
|  | Skin and subcutaneous tissue disorders | 0 (0%) | 1 (2%) | 0 (0%) | 0 (0%) | 0 (0%) | 0 (0%) |
|  | Social circumstances | 1 (2%) | 1 (2%) | 0 (0%) | 1 (1%) | 2 (2%) | 0 (0%) |
|  | Investigations | 0 (0%) | 0 (0%) | 0 (0%) | 1 (1%) | 2 (2%) | 0 (0%) |
|  | Vascular disorders | 0 (0%) | 0 (0%) | 1 (2%) | 0 (0%) | 0 (0%) | 0 (0%) |
|  | Musculoskeletal and connective tissue disorders | 1 (2%) | 0 (0%) | 1 (2%) | 0 (0%) | 0 (0%) | 0 (0%) |
|  | Respiratory, thoracic and mediastinal disorders | 0 (0%) | 1 (2%) | 0 (0%) | 1 (1%) | 1 (1%) | 0 (0%) |
|  | Metabolism and nutrition disorders | 0 (0%) | 0 (0%) | 0 (0%) | 0 (0%) | 0 (0%) | 1 (1%) |
|  | Renal and urinary disorders | 0 (0%) | 0 (0%) | 0 (0%) | 0 (0%) | 0 (0%) | 2 (2%) |
|  | Neoplasms benign, malignant and unspecified (incl cysts and polyps) | 0 (0%) | 0 (0%) | 1 (2%) | 0 (0%) | 0 (0%) | 0 (0%) |
|  | Immune system disorders | 0 (0%) | 0 (0%) | 0 (0%) | 0 (0%) | 0 (0%) | 1 (1%) |
|  | **Total (% is out of Pramipexole=128, Placebo=162)** | **4 (3%)** | **48 (38%)** | **76 (59%)** | **8 (5%)** | **49 (30%)** | **105 (65%)** |
