# Supplementary material for: A randomised double-blind, placebo-controlled trial of pramipexole in addition to mood stabilisers for patients with treatment-resistant bipolar depression (the PAX-BD study)
Source: J Psychopharmacol. 2025 Jan 20;39(2):106–20. doi: 10.1177/02698811241309622 (PMC11831867; doi:10.1177/02698811241309622)

**Figure S7**: box plots for QIDS-SR and ASRM scores for baseline, 12, 24, 36 and 48 weeks, for participants on and not on antipsychotics in the pramipexole arm


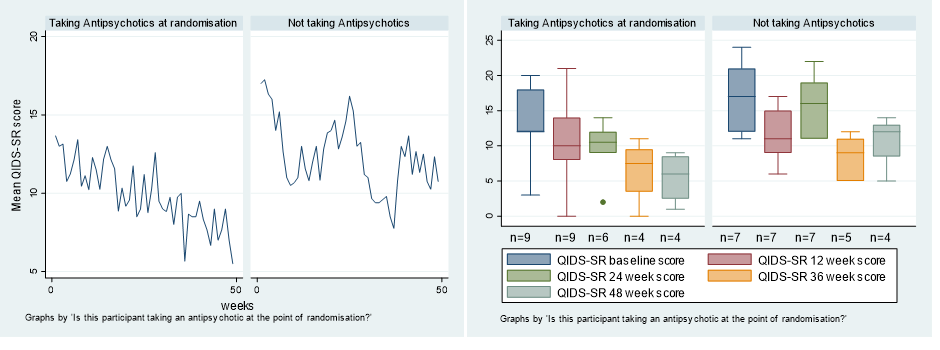

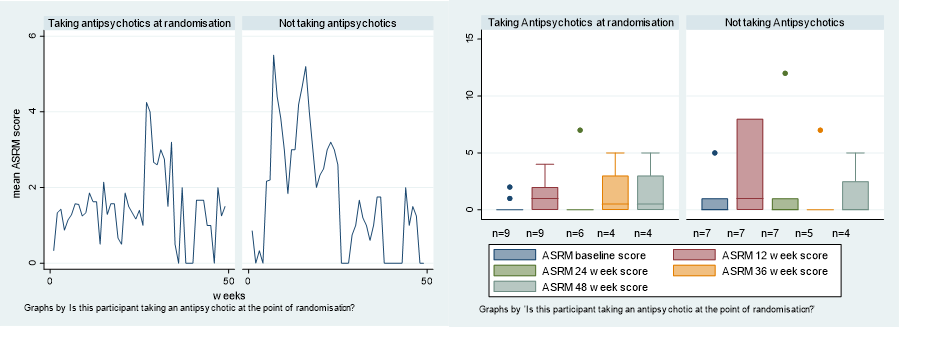

Supplement: sj-docx-7-jop-10.1177_02698811241309622 – Supplemental material for A randomised double-blind, placebo-controlled trial of pramipexole in addition to mood stabilisers for patients with treatment-resistant bipolar depression (the PAX-BD study) [file sj-docx-7-jop-10.1177_02698811241309622.docx]
