# Supplementary material for: A randomised double-blind, placebo-controlled trial of pramipexole in addition to mood stabilisers for patients with treatment-resistant bipolar depression (the PAX-BD study)
Source: J Psychopharmacol. 2025 Jan 20;39(2):106–20. doi: 10.1177/02698811241309622 (PMC11831867; doi:10.1177/02698811241309622)
Supplement: sj-docx-8-jop-10.1177_02698811241309622 – Supplemental material for A randomised double-blind, placebo-controlled trial of pramipexole in addition to mood stabilisers for patients with treatment-resistant bipolar depression (the PAX-BD study) [file sj-docx-8-jop-10.1177_02698811241309622.docx]

**Table S1** – Study sites

|  | **Date opened** | **Recruited to Pre-randomisation** | **Recruited to Randomisation** |
| --- | --- | --- | --- |
| Surrey and Borders Partnership NHS Foundation Trust | 03/12/2019 | **10** | **8** |
| Cumbria, Northumberland, Tyne & Wear NHS Trust | 28/11/2019 | **9** | **8** |
| Nottinghamshire Healthcare NHS Foundation Trust | 12/10/2020 | **6** | **6** |
| Sheffield Health & Social Care NHS Foundation Trust | 28/01/2020 | **7** | **4** |
| Cheshire and Wirral Partnership NHS Foundation Trust | 20/02/2020 | **4** | **3** |
| Tees, Esk and Wear Valleys NHS Foundation Trust | 18/02/2020 | **3** | **2** |
| Avon and Wiltshire Mental Health Partnership NHS Trust | 09/07/2021 | **2** | **2** |
| South London and Maudsley NHS Foundation Trust | 24/05/2021 | **2** | **2** |
| Kent and Medway NHS and Social Care Partnership Trust | 28/01/2020 | **2** | **1** |
| Leicestershire Partnership NHS Trust | 26/07/2021 | **2** | **1** |
| Essex Partnership University NHS Foundation Trust | 23/06/2021 | **1** | **1** |
| Oxford Health NHS Foundation Trust | 28/04/2021 | **1** | **1** |
| Devon Partnership NHS Trust | 03/02/2020 | **1** | **0** |
| Lincolnshire Partnership NHS Foundation Trust | 02/01/2020 | **1** | **0** |
| Derbyshire Healthcare NHS Foundation Trust | 20/12/2019 | **0** | **0** |
| Lancashire and South Cumbria NHS Foundation Trust | 20/01/2022 | **0** | **0** |
| NHS Lothian | 08/04/2021 | **0** | **0** |
| NHS Greater Glasgow and Clyde | 25/03/2022 | **0** | **0** |
| NHS Tayside | 17/02/2022 | **0** | **0** |
| Southwest Yorkshire Partnership NHS Foundation Trust | 11/11/2021 | **0** | **0** |
| Southwest London and St George’s Mental Health NHS Trust | 06/01/2020 | **0** | **0** |
| **Total** |  | **51** | **39** |
