# Supplementary material for: A randomised double-blind, placebo-controlled trial of pramipexole in addition to mood stabilisers for patients with treatment-resistant bipolar depression (the PAX-BD study)
Source: J Psychopharmacol. 2025 Jan 20;39(2):106–20. doi: 10.1177/02698811241309622 (PMC11831867; doi:10.1177/02698811241309622)
Supplement: sj-docx-9-jop-10.1177_02698811241309622 – Supplemental material for A randomised double-blind, placebo-controlled trial of pramipexole in addition to mood stabilisers for patients with treatment-resistant bipolar depression (the PAX-BD study) [file sj-docx-9-jop-10.1177_02698811241309622.docx]

**Table S2**: Eligibility criteria

#### Inclusion Criteria: Pre-randomisation Stage

1. Under secondary care mental health services
2. Decision made by the patient’s clinical team that a change in medication is indicated.
3. Current diagnosis of BD (type I or II).
4. Currently meeting criteria for a Major Depressive Episode (MDE) with a Quick Inventory of Depressive Symptoms, Self-Rated (QIDS-SR) score >10.
5. Suffering from TRBD defined as the failure (non-response, intolerance and/or refused/clinically not indicated) of ≥2 NICE or BAP recommended mediations for bipolar depression (quetiapine, olanzapine, lamotrigine or lurasidone) in the current episode of depression.
6. Aged 18 or over.
7. Willing and able to provide written informed consent.
8. Able to follow the trial prescription instructions and manage 8 week supplies of trial medication.
9. If female and of child-bearing potential, must have a negative pregnancy test [urine beta-human chorionic gonadotropin (β-hCG)] and required to use a highly effective contraceptive method throughout the trial.

#### Exclusion Criteria: Pre-Randomisation Stage

1. Severe substance use disorder.
2. Current psychotic symptoms.
3. History of retinal disease.
4. Current symptoms or significant concerns around cardiovascular disease.
5. History of significant renal disease.
6. Any known sensitivity to trial drug including its excipients.
7. Current or planned pregnancy during the trial period, or breastfeeding.
8. Starting specific psychotherapy from four weeks before randomisation through to week 12 post-randomisation.
9. Currently taking part in another clinical trial that would interfere with the outcomes of PAX-BD.
10. Confirmed diagnosis with potential confounding factors such as Parkinson’s disease or restless leg syndrome.
11. Significant clinical concern regarding impulse control behaviours

#### Inclusion Criteria: Randomisation Stage

1. Been in pre-randomisation stage for a minimum of 23 calendar days.
2. Currently depressed (QIDS-SR >10).
3. Minimum of two telephone/tele- or videoconference calls with a trial RA and two on-line weekly symptom ratings completed during the pre-randomisation stage
4. On mood stabilising medication (lithium, valproate, carbamazepine, lamotrigine)
5. Not on an antipsychotic. This criteria was amended during the trial to allow antipsychotics within specified dosing limits.
6. All regular psychotropic medication at a stable dose ≥4 weeks. Additionally, if taking lamotrigine, quetiapine, olanzapine or lurasidone this must have been at the current dose or higher for ≥3 months.
7. If female and of child-bearing potential, a negative urine β-hCG test and using a highly effective contraceptive method.
8. Willing and able to confirm written informed consent at the point of randomisation.

#### Exclusion Criteria: Randomisation Stage

As per pre-randomisation stage including

1. Psychotic symptoms over the preceding 4 weeks.
2. Any deterioration in physical or mental health since pre-randomisation leading to a clinical concern to proceed.
3. Electroconvulsive therapy (ECT) in the last 28 days.
4. Any concern regarding the patient’s ability to remain engaged in the trial
